# Supplementary material for: WRN helicase safeguards deprotected replication forks in BRCA2-mutated cancer cells
Source: Nat Commun. 2021 Nov 12;12:6561. doi: 10.1038/s41467-021-26811-w (PMC8590011; doi:10.1038/s41467-021-26811-w)
Supplement: Supplementary file 1 — Supplementary Information [file 41467_2021_26811_MOESM1_ESM.pdf]

Supplementary Information

WRN Helicase Safeguards Deprotected Replication Forks in *BRCA2*-Mutated Cancer Cells

Arindam Datta[1], Kajal Biswas[2], Joshua A. Sommers[1], Haley Thompson[1], Sanket Awate[1], Claudia M. Nicolae[3], Tanay Thakar[3], George-Lucian Moldovan[3], Robert H. Shoemaker[4], Shyam K. Sharan[2] and Robert M. Brosh Jr[1,]*

[1]Translational Gerontology Branch, National Institute on Aging, NIH,

Baltimore, MD 21224, USA

[2]Mouse Cancer Genetics Program, Center for Cancer Research,

National Cancer Institute, NIH, Frederick, MD 21702, USA

[3]Department of Biochemistry and Molecular Biology,

The Pennsylvania State University College of Medicine, Hershey, PA 17033, USA

[4]Chemopreventive Agent Development Research Group, Division of Cancer Prevention,

National Cancer Institute, NIH, Rockville, MD 20850, USA

*To whom correspondence should be sent:

Email: broshr@mail.nih.gov

Phone: 410-302-9558

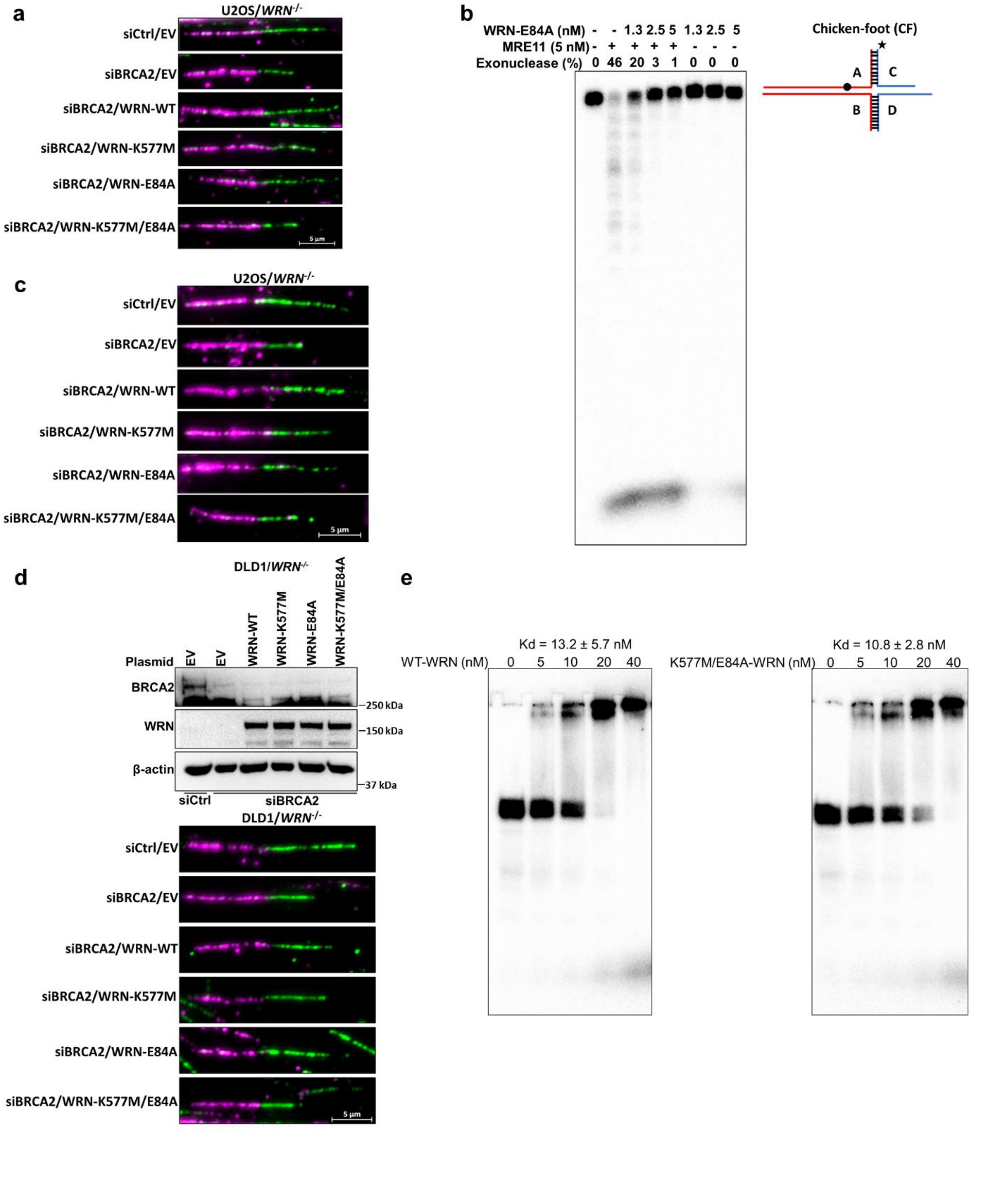

Supplementary Figure 1

Supplementary Figure 1. WRN helicase antagonizes reversed fork resection by MRE11 nuclease *in vitro* and limits hyper-degradation of stalled forks in BRCA2 depleted cells. a, Representative DNA fiber images of fork restart experiment in BRCA2-depleted U2OS/*WRN*$^{-/-}$ cells complemented with WT or catalytic dead WRN mutants. Scale bar is shown. **b**, *In vitro* nuclease assay showing reduced 3´-5´ MRE11 exonuclease activity on CF DNA substrates in the presence of WRN-E84A. CF substrate was preincubated with recombinant WRN-E84A for 10 min as described in Methods section, followed by 50 min incubation with recombinant MRE11. Reaction products were deproteinated, heated at 95°C for 5 min in formamide loading dye and resolved by denaturing 12% polyacrylamide gel electrophoresis with 0.5X TBE and 8 M urea. Gel was exposed to a phosphorscreen and imaged using a Typhoon FLA 9500 imager. Percent exonuclease was calculated as the radioactivity found below the intact oligo band as a percentage of the total radioactivity detected in each lane. Mononucleotide at the bottom of the gel was included as part of the total radioactivity but not as exonuclease activity. Star indicates radiolabeled ([γ-^{32}P] ATP) at 5´ DNA end. Experiment was repeated three (*n*=3) times with similar results. **c**, Representative DNA fiber images of fork protection experiment in BRCA2-depleted U2OS/*WRN*$^{-/-}$ cells complemented with WT or catalytic dead WRN mutants. **d**, Immunoblots showing BRCA2 and WRN protein levels in control or BRCA2 siRNA-treated DLD1/*WRN*$^{-/-}$ cells transfected with either control empty vector (EV) or with WRN-WT, WRN-K577M, WRN-E84A, or WRN-K577M/E84A expression plasmids. β-actin was used as loading control. Representative DNA fiber images of fork protection experiment in BRCA2-depleted DLD1/*WRN*$^{-/-}$ cells complemented with WT or catalytic dead WRN mutants are shown. **e**, Electrophoretic mobility shift assay (EMSA) to assess binding of WT or helicase-exonuclease dead (K577M/E84A) WRN to reversed fork DNA substrate. Autoradiogram showing representative EMSA assay using 2 nM reversed fork ("chicken-foot") substrate and indicated concentrations of WT or K577M/E84A WRN proteins and 2 mM ATPγS as described in Methods. Reactions were resolved on 5% 1X TBE gels, exposed to a phosphorscreen and imaged using a Typhoon FLA 9500 imager. Dissociation constant (Kd) values for substrate binding were calculated by graphing the log of the WRN concentration vs. the log of the fraction bound/fraction unbound ratio and calculating the anti-log of the y-value at x = 0. Experiment was repeated three (*n*=3) times with similar results. Micrographs (**a**, **c**, and **d**) and immunoblots (**d**) are representative of at least two independent experiments with similar results. Source data are provided as a Source Data file.

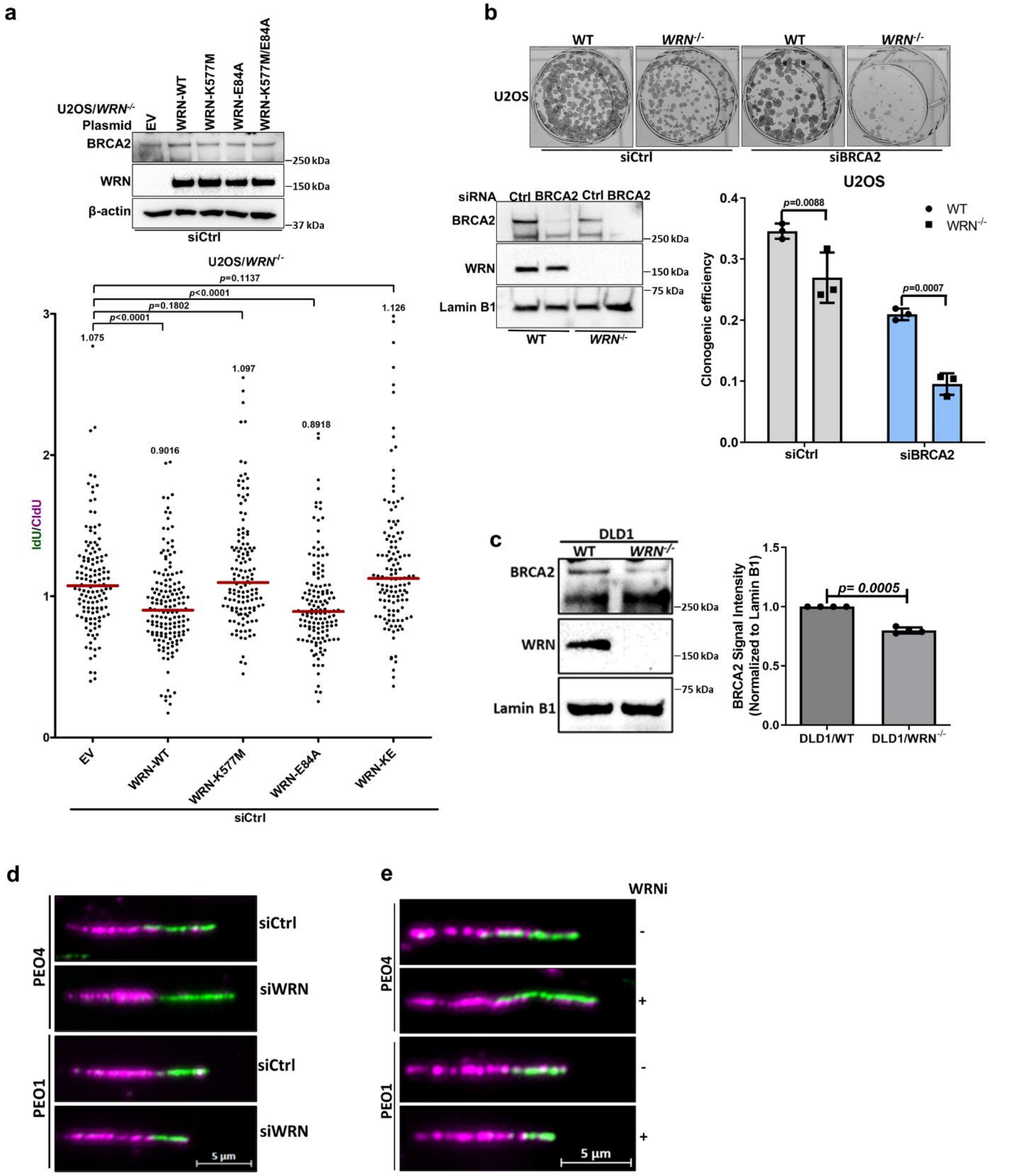

Supplementary Figure 2

Supplementary Figure 2. WRN helicase promotes limited nucleolytic processing in BRCA2-proficient cells. a, **(**Upper panel) Immunoblots showing BRCA2 and WRN protein levels in control siRNA-treated U2OS/*WRN*$^{-/-}$ cells transfected with either control empty vector (EV) or with WRN-WT, WRN-K577M, WRN-E84A, or WRN-K577M/E84A expression plasmids. (Lower panel) Fork stability assay to assess HU-stalled fork processing by WRN WT or catalytic dead mutants under BRCA2 wild type condition. Scatter dot plot showing IdU to CldU tract length ratios in individual experimental conditions. Representative of n=2 independent experiments; p-values (p<0.0001, p=0.1802, p<0.0001, p=0.1137) were derived from $n \geq 130$ DNA fibers using two-tailed Mann–Whitney test. Horizontal red bars indicate median of IdU/CldU ratios; median IdU/CldU values are indicated. **b**, Colony formation ability of WT or *WRN*$^{-/-}$ U2OS cells upon BRCA2 knockdown. WT or *WRN*$^{-/-}$ U2OS cells transfected with either control or BRCA2 siRNA (80 nM) were plated on a 6-well plate at 1000 cells/well 72 h post transfection and allowed to form colonies for 12 days. Representative images of colonies are shown. Bar graphs showing clonogenic efficiency (number of colonies formed/total number of cells seeded) of control or BRCA2-depleted U2OS/WT and U2OS/*WRN*$^{-/-}$ cells. Data represent mean ± SD of three (n=3) independent experiments. BRCA2 knockdown was verified by Western blotting. Lamin-B1 was used as loading control. Two-way ANOVA with Bonferroni post-test (n=3); p-values (p=0.0088, p=0.0007) are indicated**. c**, Immunoblots showing BRCA2 protein level in WT and *WRN*$^{-/-}$ isogenic DLD1 cells. Bar graph represents BRCA2 signal intensity normalized to Lamin B1. Data represent mean ± SD (n=4). Two-tailed Student's t-test, p value (p=0.0005) is shown. **d**, Representative DNA fiber images of fork protection experiment in PEO4 and PEO1 cells upon WRN knockdown. Scale bar is shown. **e**, Representative DNA fiber images of fork protection experiment in PEO4 and PEO1 cells exposed to WRNi. Micrographs are representative of three (**d**) or two (**e**) independent experiments with similar results. Source data are provided as a Source Data file.

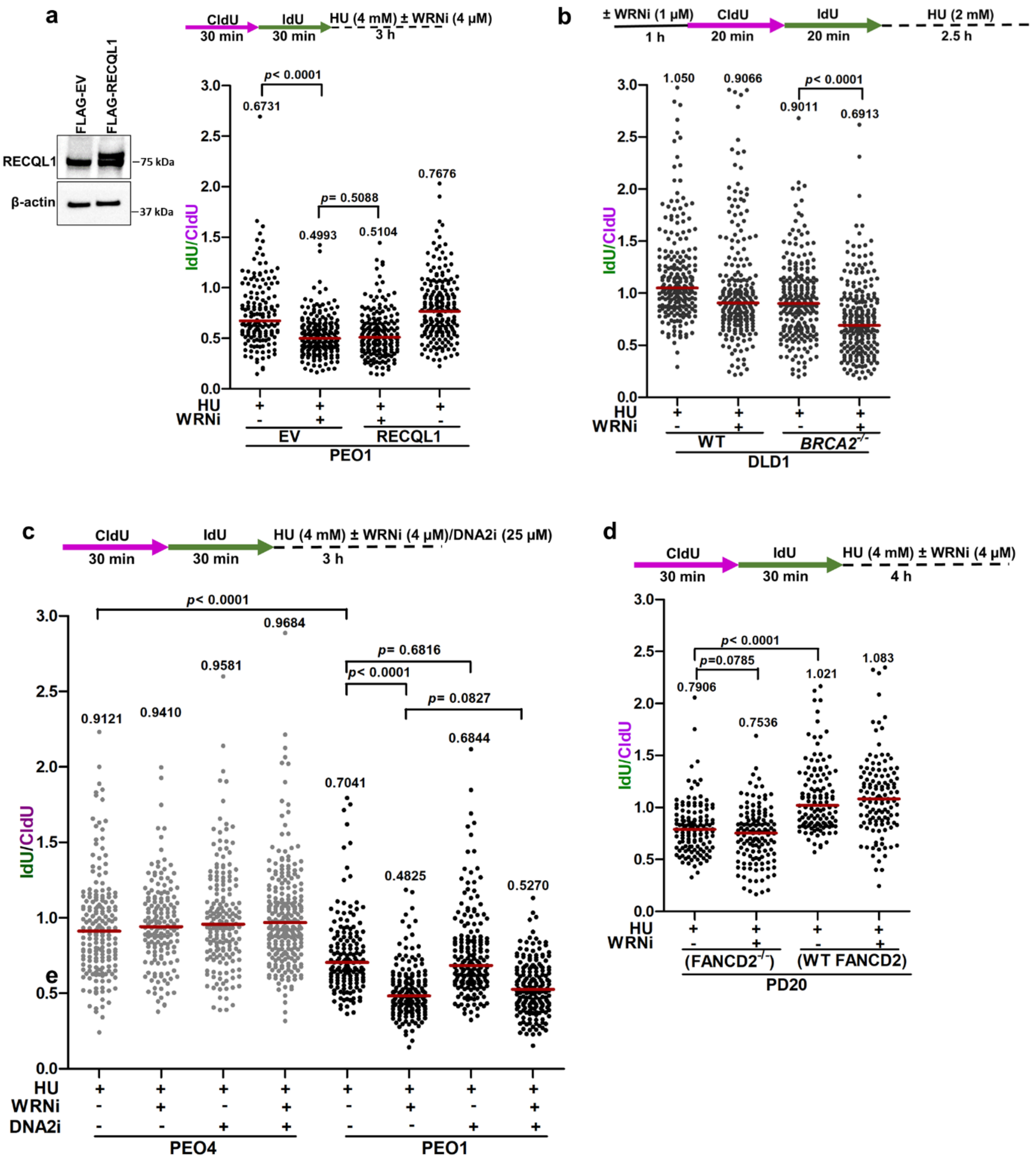

Supplementary Figure 3

Supplementary Figure 3. WRN helicase inhibition triggers fork degradation in BRCA2-deficient cancer cells. a, Fork stability assay in PEO1 cells overexpressing RECQL1 following WRNi treatment. Cells were transfected with 2 μg of either empty vector (EV) or FLAG-tagged RECQL1 expression plasmid and subjected to fork stability assay 48 h post transfection. Scatter dot plot represents IdU/CldU ratios in each condition. Representative of n=2 independent experiments; p-values (p<0.0001, p=0.5088) were derived from $n \geq 150$ DNA fibers using two-tailed Mann–Whitney test. RECQL1 overexpression was confirmed by Western blotting. β-actin was used as loading control. **b**, Fork stability assay performed in WT and $BRCA2^{-/-}$ DLD1 cells exposed to WRNi. Cells were optionally pretreated with WRNi and subjected to fork stability assay as depicted. Scatter dot plot represents quantification of the results obtained. Representative of n=2 independent experiments; p-values (p<0.0001) were derived from $n \geq 225$ DNA fibers using two-tailed Mann–Whitney test. **c,** Fork stability assay in PEO4 and PEO1 cells exposed to WRNi or DNA2 inhibitor (DNA2i). Following CldU and IdU labeling, cells were subjected to 4 mM HU treatment along with WRNi (4 μM) or DNA2i (25 μM) for 3 h. Scatter dot plot showing IdU/CldU ratios in individual experimental conditions. Representative of n=2 independent experiments; p-values (p<0.0001, p<0.0001, p=0.6816, p=0.0827) were derived from $n \geq 150$ DNA fibers using two-tailed Mann–Whitney test. **d**, Fork stability assay in isogenic FANCD2-deficient and FANCD2-corrected PD20 cells following WRN helicase inhibition. Scatter dot plot represents IdU/CldU ratios in individual samples. Representative of n=2 independent experiments; p-values (p=0.0785, p<0.0001) were derived from $n \geq 125$ DNA fibers using two-tailed Mann–Whitney test. Horizontal red bars indicate median of IdU/CldU ratios; purple and green colors indicate CldU and IdU labeling, respectively; Median IdU/CldU values are indicated. Source data are provided as a Source Data file.

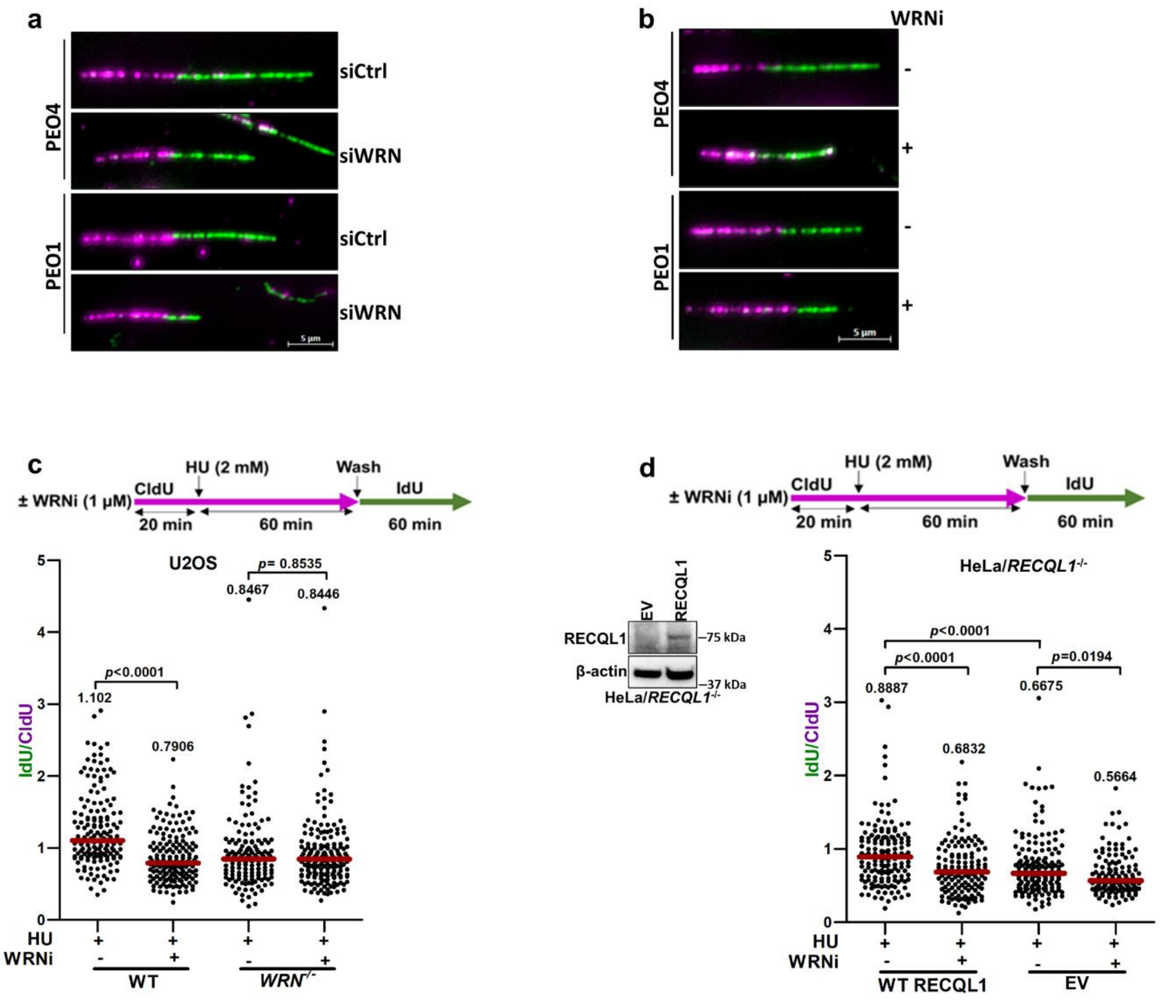

Supplementary Figure 4

Supplementary Figure 4. Defective replication restart induced by WRNi is WRN-dependent. **a** and **b**, Representative DNA fiber images of fork restart experiments in PEO4 and PEO1 cells upon WRN knockdown (a) or WRN helicase inhibition (b). Scale bars are shown. Micrographs are representative of two independent experiments with similar results. **c**, Fork restart experiment in WT and *WRN*$^{-/-}$ U2OS cells exposed to WRNi. Schematic of the fiber experiment is shown. Scatter dot plot represents quantification of the results. Representative of n=2 independent experiments; p-values (p<0.0001, p=0.8535) were derived from $n \geq 150$ DNA fibers using two-tailed Mann–Whitney test. **d**, Fork restart experiment in *RECQL1*$^{-/-}$ HeLa cells carrying control empty vector (EV) or complemented with WT RECQL1. Immunoblots showing RECQL1 protein level in RECQL1 complemented HeLa/*RECQL1*$^{-/-}$ cells. β-actin was used as loading control. Graphical quantification of the results obtained from the fork restart experiment is shown. Representative of n=2 independent experiments; p-values (p<0.0001, p<0.0001, p=0.0194) were derived from $n \geq 125$ DNA fibers using two-tailed Mann–Whitney test. In **c** and **d**, horizontal red bars indicate median of IdU/CldU ratios; purple and green colors indicate CldU and IdU labeling, respectively; Median IdU/CldU values are indicated. Source data are provided as a Source Data file.

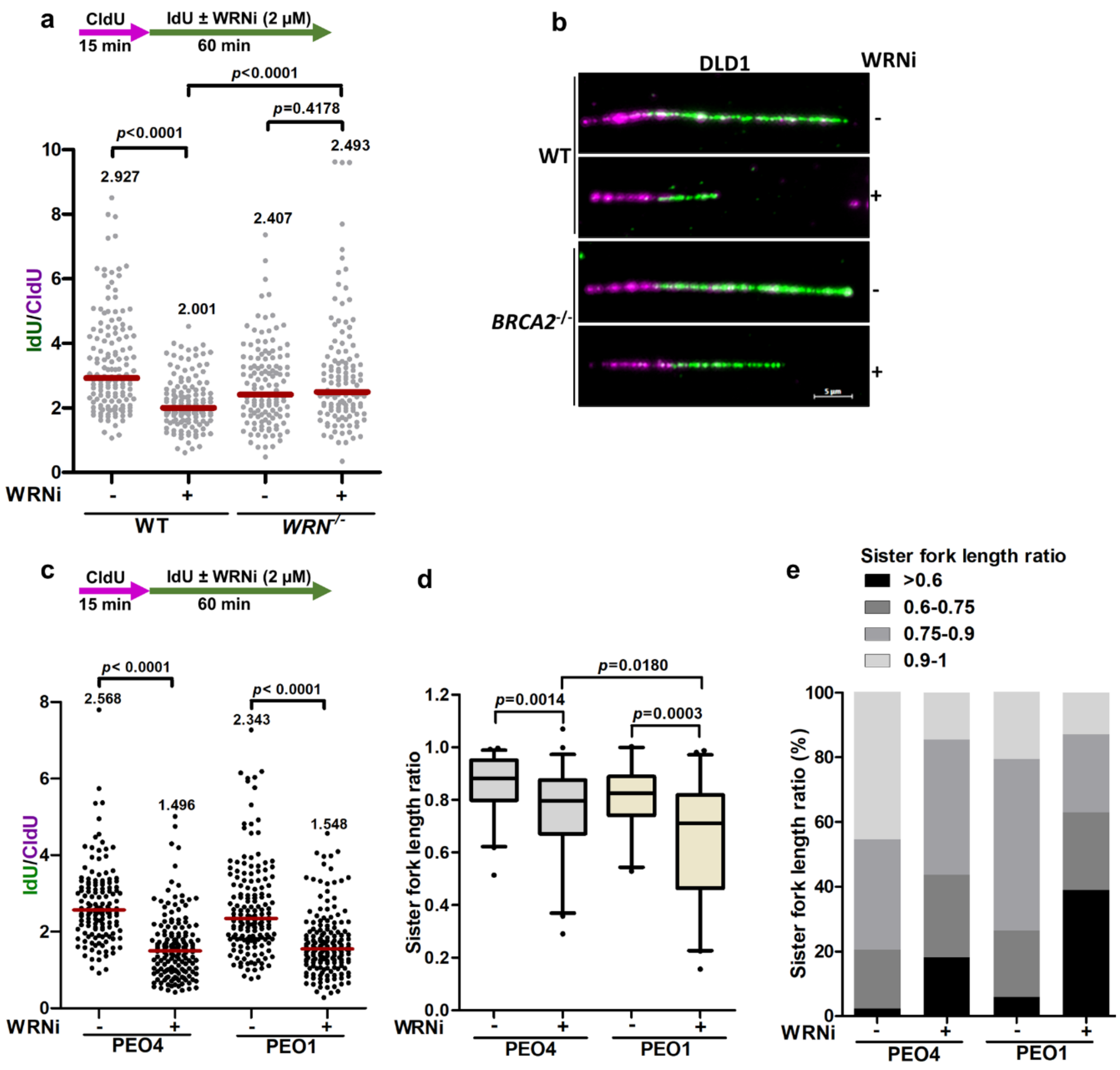

Supplementary Figure 5

Supplementary Figure 5. WRN helicase inhibition promotes replication fork stalling. a, (Upper panel) Schematic showing fiber labeling strategy for assessing replication fork progression in WT and isogenic *WRN*$^{-/-}$ U2OS cells upon WRNi treatment. Asynchronously growing cells were sequentially labeled with CldU and IdU for 15 and 60 min, respectively. WRNi (2 µM) was added concomitantly with the IdU labeling. (Lower panel) Graphical quantification of IdU/CldU tract ratios in WT and *WRN*$^{-/-}$ U2OS cells treated or untreated with WRNi. Representative of n=2 independent experiments; p-values (p<0.0001, p<0.0001, p=0.4178) were derived from $n \geq 120$ DNA fibers using two-tailed Mann–Whitney test. **b**, Representative DNA fiber images of fork progression experiment in WT and *BRCA2*$^{-/-}$ DLD1 cells exposed to WRNi. Scale bar is shown. **c**, Replication fork progression in PEO4 and PEO1 cells upon WRN helicase inhibition. Representative of n=3 independent experiments; p-values (p<0.0001, p<0.0001) were derived from $n \geq 140$ DNA fibers using two-tailed Mann–Whitney test. **d**, Quantitation of fork asymmetry from experiment described in **c**. Bounds of box represent 25th-75th percentile, middle horizontal lines show the median, whiskers indicate 5th–95th percentiles. Representative of n=3 independent experiments; p-values (p=0.0014, p=0.0180, p=0.0003) were derived from $n \geq 30$ bidirectional forks using two-tailed Mann–Whitney test. **e**, Bar graph showing percent distribution of sister fork length ratios. In **a** and **c**, horizontal red bars indicate median of IdU/CldU ratios; purple and green colors indicate CldU and IdU labeling, respectively; Median IdU/CldU values are indicated. Source data are provided as a Source Data file.

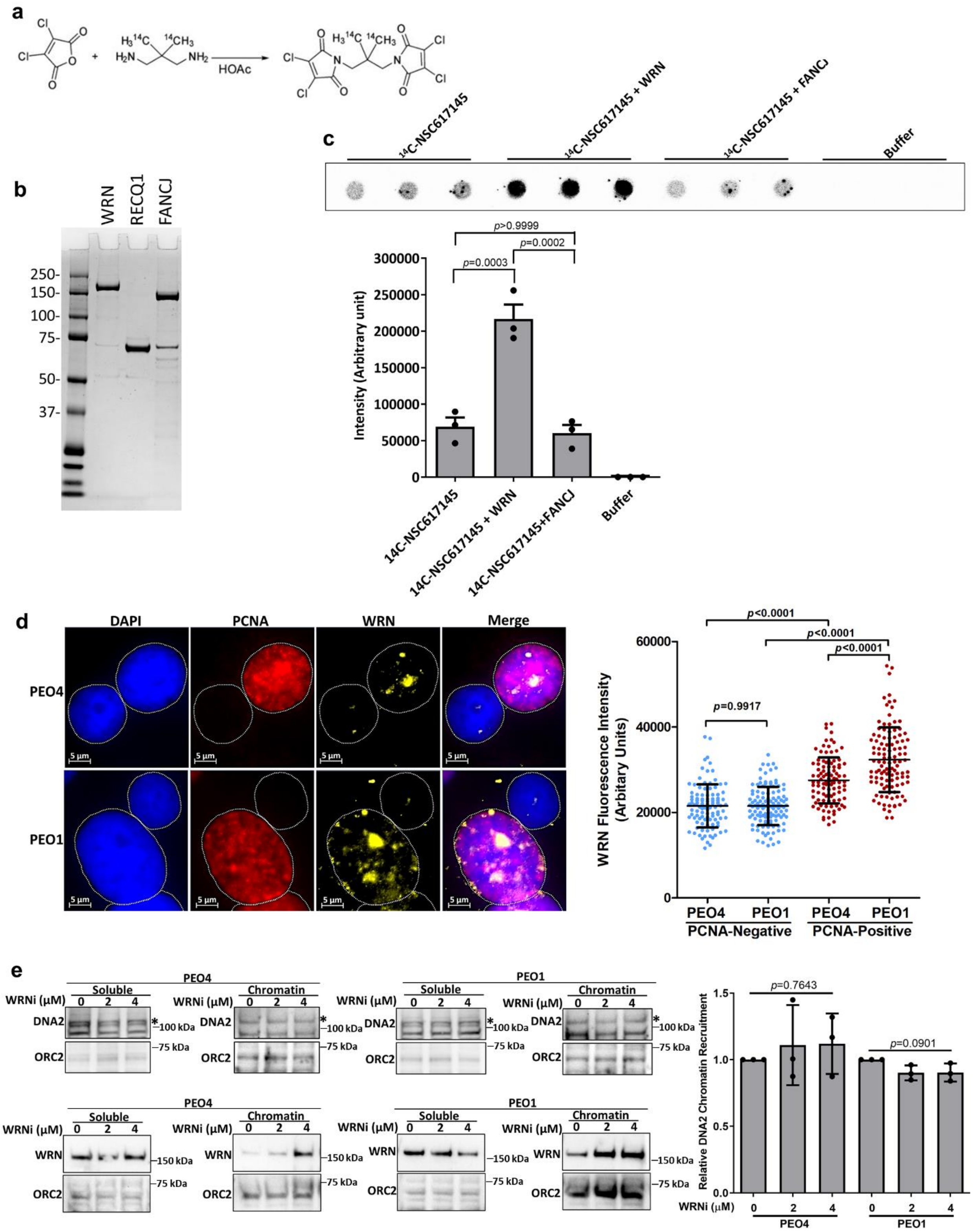

Supplementary Figure 6

Supplementary Figure 6. Selective binding and chromatin sequestration of WRN by NSC617145. a, The synthesis reaction of 14C-NSC617145. **b**, SDS-PAGE analysis of purified full length recombinant WRN, RECQL1 and FANCJ proteins. Immunoblot is representative of two independent experiments with similar results. **c**, Dot blots showing *in vitro* binding of radiolabeled ^{14}C-NSC617145 (10 µM) to purified recombinant WRN (300 nM) or FANCJ (300 nM). (Lower panel) Bar graph represents quantitation of the data obtained from the drug binding experiment. Data represent mean ± SEM of three independent experiments. One-way ANOVA with Bonferroni's multiple comparison test; *p*-values (*p*=0.0003, *p*>0.999, *p*=0.0002) are indicated. **d,** WRN immunofluorescence in PEO4 and PEO1 cells co-stained with PCNA to mark replicating cells. Cells were counterstained with DAPI to mark the nuclei. Scatter dot plot represents WRN immunofluorescence intensity (*n*≥100 cells) in PCNA-negative and PCNA-positive PEO4 and PEO1 cells. Data represent mean ± SD of *n* ≥100 nuclei examined under each condition over *n*=2 independent experiments; *p* values (*p*=0.9917, *p*<0.0001, *p*<0.0001, *p*<0.0001) are indicated; two-tailed unpaired t-test. **e**, Western blot analysis of DNA2 and WRN in soluble or chromatin fractions prepared from PEO4 and PEO1 cells treated with indicated doses of WRNi for 1 h. ORC2 serves as a positive marker for chromatin fractions. Star indicates a specific band of DNA2. Bar graph represents DNA2 signal intensity normalized to ORC2 in chromatin fractions and represented as relative fold change over untreated condition (set as one). Data represent mean ± SD (*n*=3); *p*-values (*p*=0.7643, *p*=0.0901) are indicated; One-way ANOVA with Bonferroni's multiple comparison test. Source data are provided as a Source Data file.

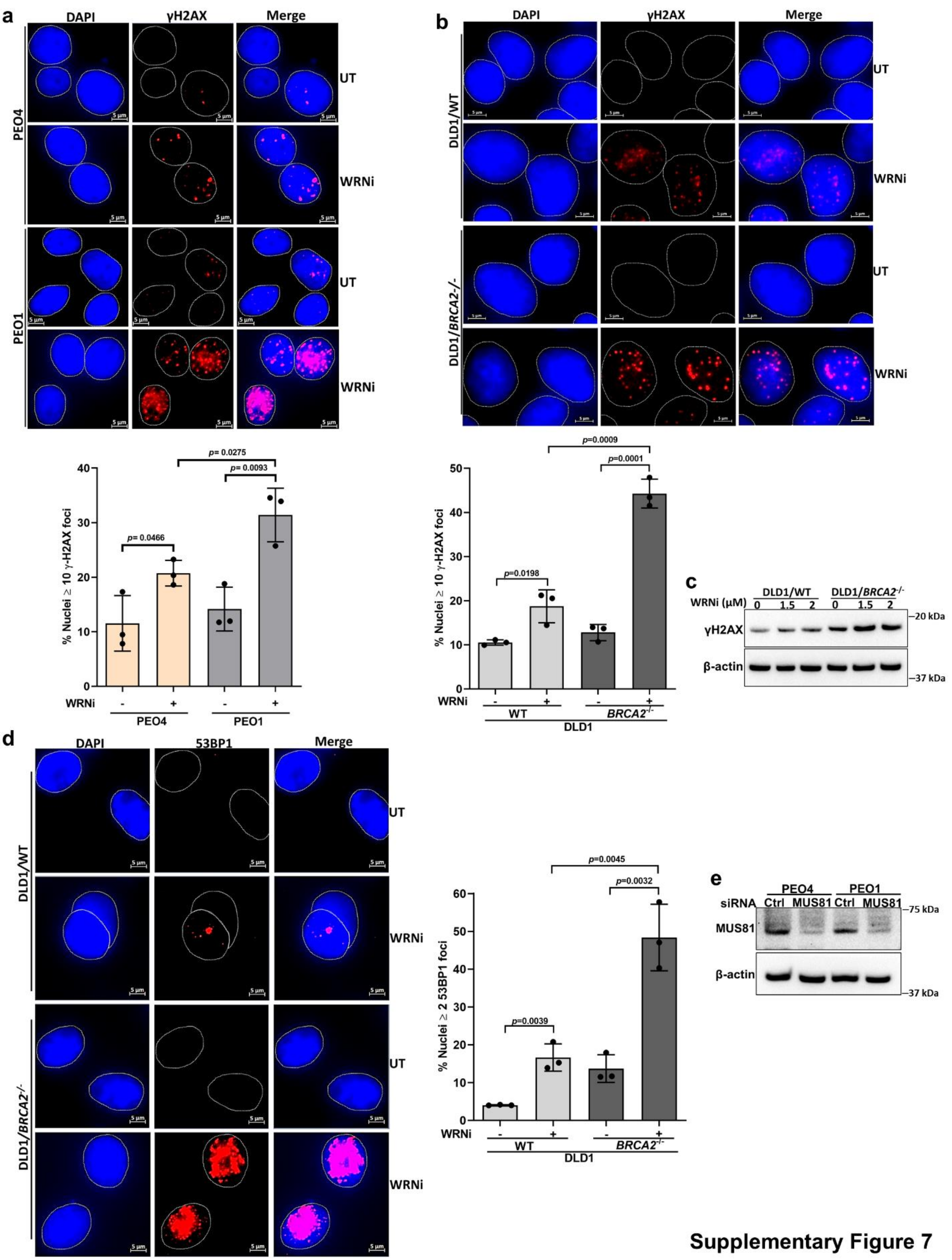

Supplementary Figure 7

Supplementary Figure 7. DNA damage accumulation in *BRCA2*-deficient cells upon WRN helicase inhibition. a, (Upper panel) Representative immunofluorescent images of γH2AX positive nuclei in PEO1 and PEO4 cells treated or untreated (UT) with 1.5 µM of WRNi (NSC617145) for 48 h. (Lower panel) Graphical quantification of %γH2AX positive nuclei in cells as described above. **b**, (Upper panel) Representative images of immunofluorescence staining for γH2AX in WT and *BRCA2*$^{-/-}$ DLD1 cells treated with 1.5 µM of WRNi (NSC617145) for 48 h. (Lower panel) Bar graph showing relative percentages of γH2AX positive nuclei in individual genotypes upon WRNi treatment. **c**, Western blots showing γH2AX protein levels in WT and *BRCA2*$^{-/-}$ DLD1 cells untreated or treated with the indicted doses of WRNi for 48 h. β-actin was used as loading control. **d**, (Left panel) Representative images of WT and *BRCA2*$^{-/-}$ DLD1 cells stained for 53BP1 upon WRNi (1.5 µM) treatment for 48 h. (Right panel) Bar graph showing relative quantification of %53BP1 foci positive WT or *BRCA2*$^{-/-}$ cells upon WRNi treatment. Scale bars are shown. In **a**, **b** and **d**, data represent mean ± SD of three independent experiments. *p* values are indicated; two-tailed unpaired t-test. **e**, Western blots showing MUS81 knockdown level in PEO4 and PEO1 cells transfected with control or MUS81 siRNA (80 nM). Total cell lysates were prepared from siRNA transfected cells 72 h post transfection and subjected to Western blotting. β-actin was used as loading control. Western blots were repeated independently three (**c**) or two (**e**) times with similar results. Source data are provided as a Source Data file.

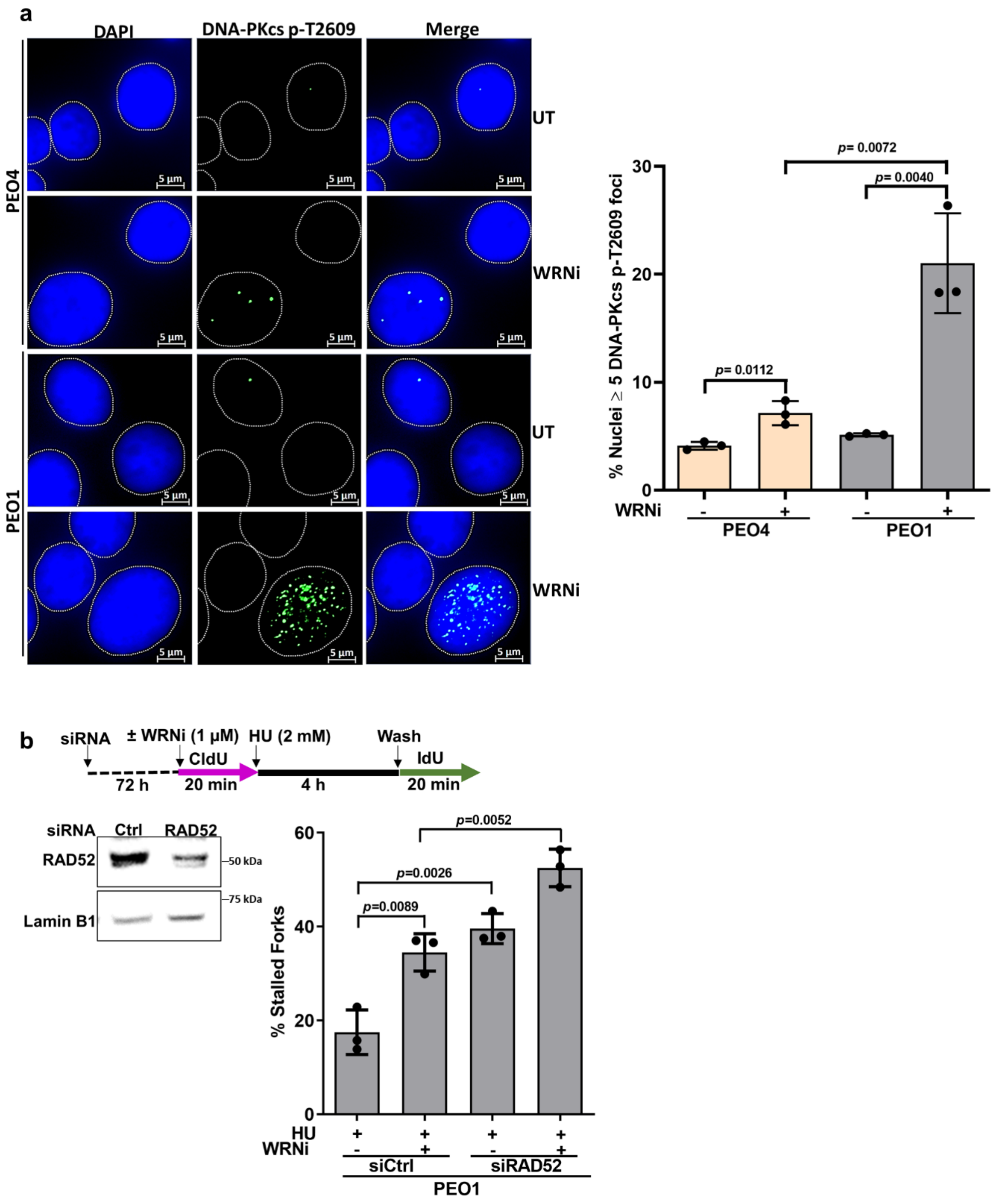

Supplementary Figure 8

Supplementary Figure 8. WRN helicase inhibition triggers NHEJ in *BRCA2*-mutated cancer cells. a, (Left panel) Immunofluorescence images showing phospho-Thr^{2609}-DNA-PKc foci in PEO1 and PEO4 cells treated with 1.5 µM of WRNi (NSC617145) for 48 h. (Left panel) Bar graphs showing % nuclei positive for phospho-Thr^{2609}-DNA-PKc foci upon WRNi treatment. Data represent mean ± SD of three independent experiments; two-tailed unpaired t-test; *p* values are indicated. **b**, Analysis of fork restart in WRNi-treated PEO1 cells upon RAD52 depletion. Cells were transfected with either control or RAD52 siRNA (80 nM) and fork restart experiment was performed 72 h post-transfection. Schematic of the fiber experiment is shown. RAD52 knockdown was verified by immunoblotting. Lamin B1 was used as loading control. Bar graphs represent percentage of stalled forks determined as percentage of only red labeled fibers among the total red labeled (only red + red-green fibers) tracts. Data represent mean ± SD of *n* = 3 independent experiments. *p* values are indicated; two-tailed unpaired t-test. Source data are provided as a Source Data file.

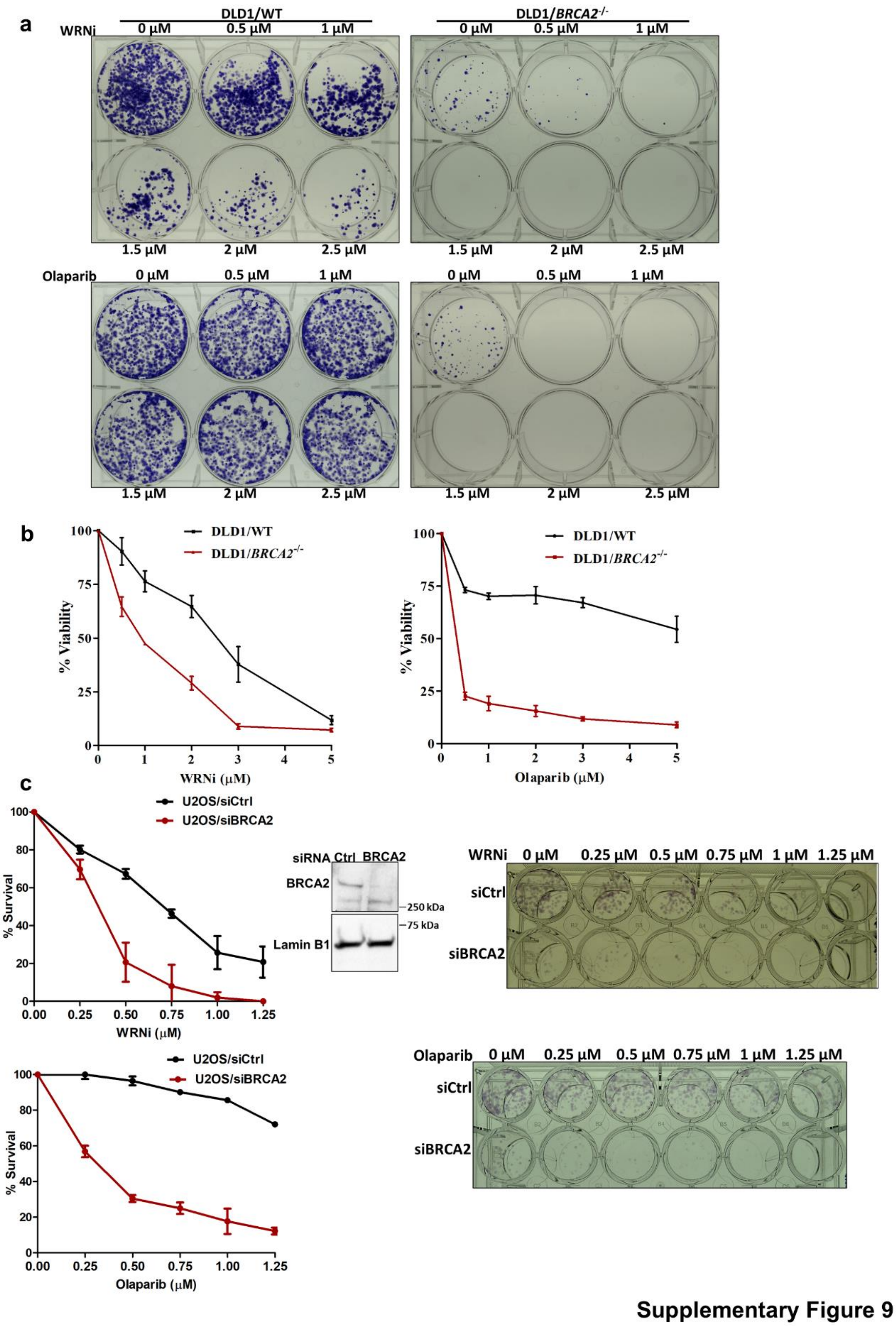

Supplementary Figure 9

Supplementary Figure 9. BRCA2-deficiency sensitizes cancer cells to WRN helicase inhibition. **a**, Representative images of colonies formed by WT and *BRCA2*$^{-/-}$ DLD1 cells upon WRNi (upper panel) or olaparib (lower panel) treatment. **b**, Dose-dependent cell viability assay in DLD1/WT and DLD1/*BRCA2*$^{-/-}$ cells upon WRNi (left panel) or olaparib (right panel) treatment. Cells (1,500 cells in 100 µl complete cell culture medium) were seeded on a 96-well plate and grown overnight before drug treatment. Cells were treated with the indicated doses of WRNi or olaparib for 72 h and subjected to WST1 assay. **c**, Clonogenic survival of WT or BRCA2-depleted U2OS cells exposed to increasing concentrations of WRNi or olaparib. U2OS cells transfected with either control or BRCA2 siRNA (80 nM) were plated on a 24-well plate at 500 cells/well 72 h post transfection. Cells were treated with the indicated doses of WRNi or olaparib 4 h after cell seeding and allowed to form colonies in presence of WRNi or olaparib for 10 days. Representative images of colonies are shown. Data represent mean ± SD of three independent biological experiments. Knockdown of BRCA2 was verified by Western blotting. Lamin-B1 serves as loading control in immunoblots. Source data are provided as a Source Data file.

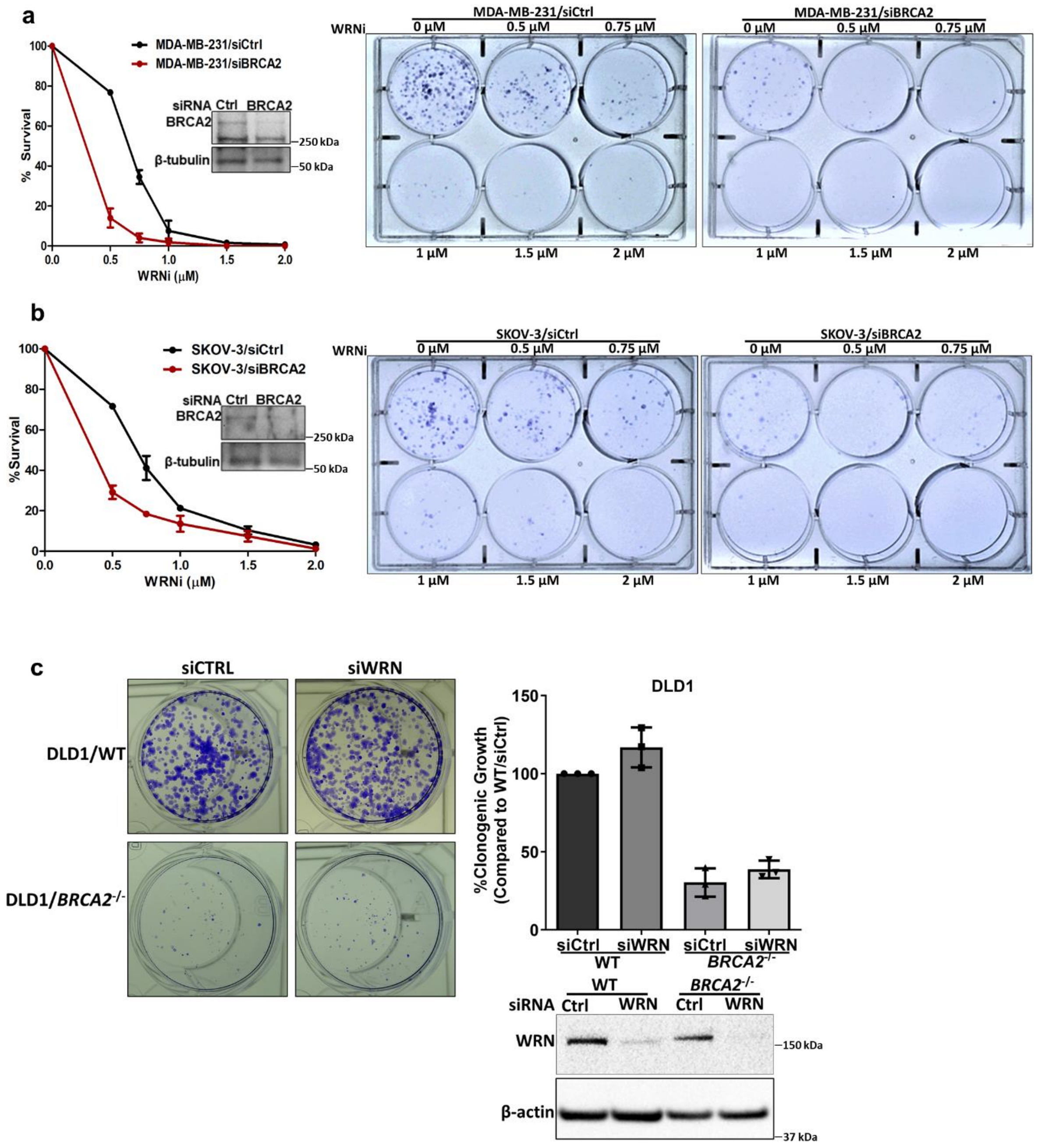

Supplementary Figure 10

Supplementary Figure 10. Clonogenic survival of *BRCA2*-deficient cancer cells upon WRN helicase inhibition or genetic depletion of WRN. (a and **b)**, Clonogenic survival of WT or BRCA2-depleted MDA-MB-231 **(a)** and SKOV-3 **(b)** cells upon WRN helicase inhibition. MDA-MB-231 and SKOV-3 cells transfected with either control or BRCA2 siRNA (80 nM) were plated on a 6-well plate at 500 cells/well 72 h post transfection. Cells were treated with the indicated doses of WRNi 4 h after cell seeding and grown in presence of WRNi for 14 days. Representative images of colonies are shown. **c**, Clonogenic potential of DLD1/WT and DLD1/*BRCA2*$^{-/-}$ cells upon WRN knockdown. WT and *BRCA2*$^{-/-}$ cells transfected with either control or WRN siRNA (80 nM) were plated on a 12-well plate at 500 cells/well 72 h post transfection and grown for 10 days. Representative images of colonies are shown. Bar graph represents clonogenic efficiency of DLD1/WT and DLD1/*BRCA2*$^{-/-}$ cells transfected with either control or WRN siRNA. Data represent mean ± SD of three independent biological experiments. Knockdown of BRCA2 (**a** and **b**) or WRN (**c**) was verified by Western blotting. β-tubulin and β-actin were used as loading controls in immunoblots. Source data are provided as a Source Data file.

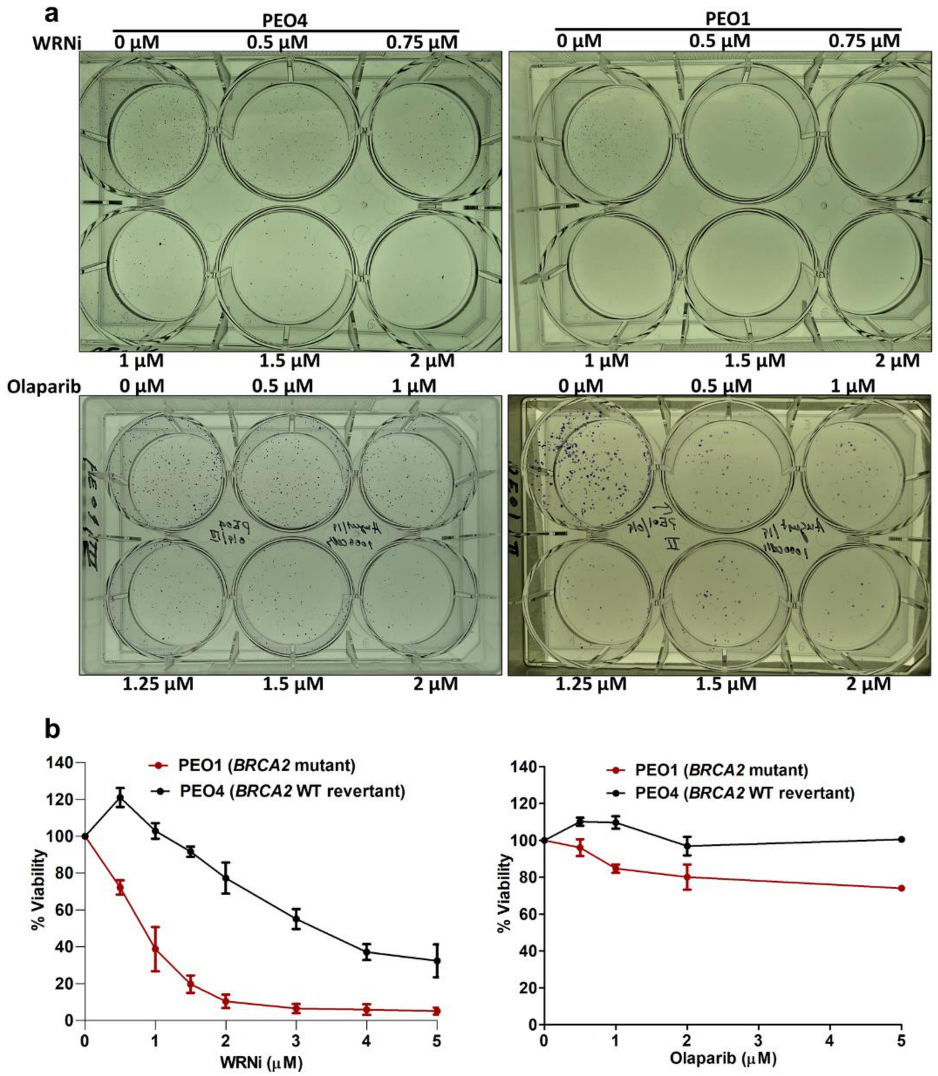

Supplementary Figure 11

Supplementary Figure 11. *BRCA2*-mutated cancer cells are hypersensitive to WRNi. a, Representative images of colonies formed by PEO1 and PEO4 cells upon NSC617145 (WRNi) (upper panel) or olaparib (lower panel) treatment. **b**, Line graphs showing relative viability of PEO1 and PEO4 cells exposed to increasing doses of WRNi (left panel) or olaparib (right panel). Data represent mean ± SD of two (n=2) independent biological experiments. Cells (1,500 cells in 100 µl complete cell culture medium) were seeded on a 96-well plate and grown overnight before drug treatment. Cells were treated with the indicated doses of WRNi or olaparib for 72 h and relative viability was determined by WST1 assay. Source data are provided as a Source Data file.

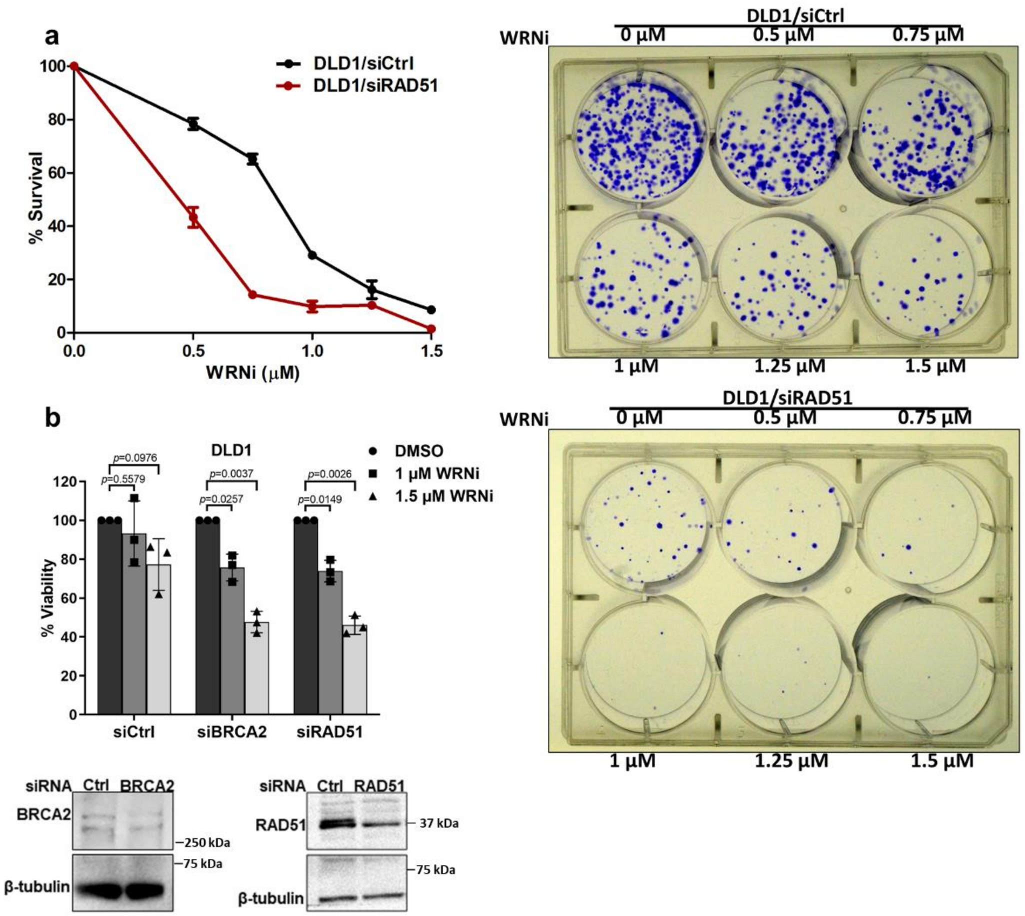

Supplementary Figure 12

Supplementary Figure 12. RAD51-depleted cells are hypersensitive to WRN helicase inhibition. a, Clonogenic survival of control or RAD51 knocked down DLD1 cells upon WRNi treatment. DLD1 cells transfected with either control or RAD51 siRNA (80 nM) were plated on a 6-well plate at 500 cells/well 72 h post transfection. Cells were treated with the indicated doses of WRNi 4 h after cell seeding and grown in presence of the drug for 16 days. Representative images of colonies are shown. **b**, Bar graph showing relative viability of DLD1 cells exposed to the indicated doses of WRNi (NSC617145) upon siRNA mediated knockdown BRCA2 or RAD51. DLD1 cells transfected with BRCA2 or RAD51 siRNA (80 nM) were plated on a 96-well plate (1,500 cells per well) 24 h post-transfection. After 48 h of siRNA transfection, cells were treated with the indicated doses of NSC617145 for 48 h and relative cell viability was measured by WST1 assay. Knockdown of BRCA2 and RAD51 was verified by Western blotting. β-tubulin was used as loading control in immunoblots. For BRCA2 blot, β-tubulin control derived from the same experiment was run on a different gel. Data represent mean ± SD of three independent experiments. Two-tailed Student's t-test; *p* values (p=0.5579, p=0.0976, p=0.0257, p=0.0037, p=0.0149, p=0.0026) are indicated. Source data are provided as a Source Data file.

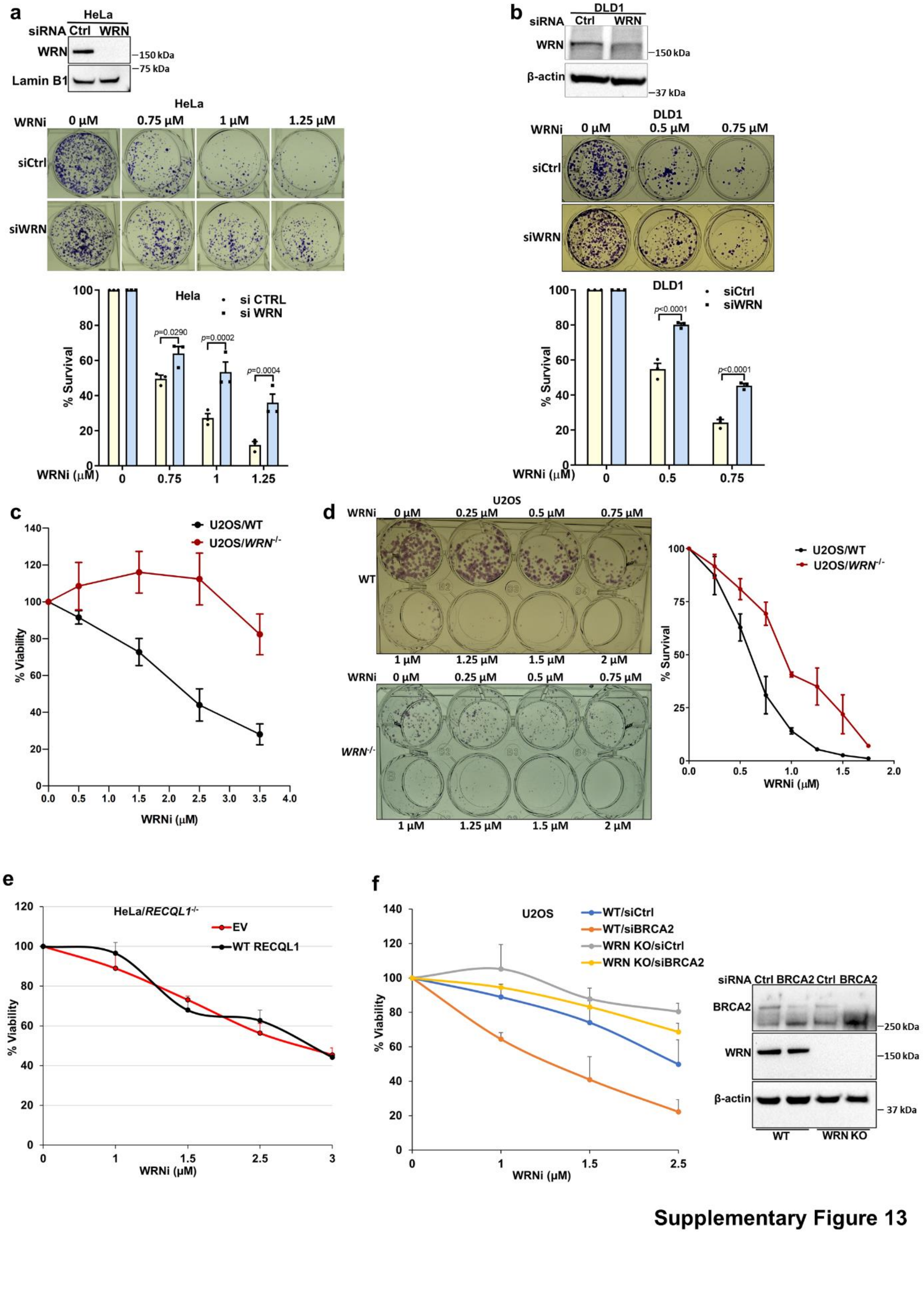

Supplementary Figure 13

Supplementary Figure 13. Cytotoxicity of NSC617145 is WRN-dependent. (a and **b)**, Representative images of colonies formed by control or WRN-depleted HeLa (**a**) and DLD1 (**b**) cells exposed to indicated doses of WRNi. HeLa cells transfected with either control or WRN siRNA (80 nM) were plated on a 6-well plate at 1,000 cells/well 72 h post transfection. Cells were treated with the indicated doses of WRNi 4 h after cell seeding and grown in presence of WRNi for 12 days. DLD1 cells transfected with either control or WRN siRNA (80 nM) were seeded on a 12-well plate at 500 cells/well 72 h post transfection and subsequently treated with the indicated doses of WRNi 4 h after cell seeding. Cell were grown in presence of WRNi for 10 days. Bar graphs represent colony survival relative to untreated condition (set as 100%) in control or WRN depleted cells. Data represent mean ± SEM of three independent experiments. Two-way ANOVA with Bonferroni post-test; *p* values in **a** (p=0.0290, p=0.0002, p=0.0004) and **b** (p<0.0001, p<0.0001) are indicated. Knockdown of endogenous WRN was verified by Western blotting. Lamin B1 and β-actin were used as loading controls. **(c** and **d)**, Line graphs showing relative viability (**c**) and colony survival (**d**) of WT and $WRN^{-/-}$ U2OS cells exposed to indicated doses of WRNi. For cell viability assay, WT and $WRN^{-/-}$ U2OS cells (1,400 cells in 100 µl complete cell culture medium) were seeded on a 96-well plate and grown overnight before drug treatment. Cells were treated with the indicated doses of WRNi for 48 h and relative viability was determined by WST1 assay. For colony survival experiment, WT (500 cells/well) or $WRN^{-/-}$ (1,000 cells/well) U2OS cells were seeded on a 12-well plate and subsequently treated with the indicated doses of WRNi 4 h after cell seeding. Cell were grown in presence of WRNi for 10 days. Representative images of colonies are shown. **e**, WST1 cytotoxicity assay to assess WRNi sensitivity of HeLa/$RECQL1^{-/-}$ cells carrying control empty vector (EV) or complemented with WT RECQL1. Cells (1,500 cells in 100 µl complete cell culture medium) were seeded on 96 well plate and grown overnight before drug treatment. Cells were treated with the indicated doses of WRNi for 72 h and relative cell viability was determined by WST1 assay. Data represent mean ± SD of three (n=3) independent experiments. **f**, WST1 cytotoxicity assay in control or BRCA2-depleted WT and WRN KO U2OS cells exposed to indicated doses of WRNi. WT and WRN KO cells transfected with control or BRCA2 siRNA (80 nM) were plated on a 96-well plate (1,400 cells per well) 24 h post-transfection. After 48 h of siRNA transfection, cells were treated with the indicated doses of WRNi for an additional 48 h and relative cell viability was measured by WST1 assay. Data represent mean ± SD of three (n=3) independent experiments.

BRCA2 knockdown was verified by Western blotting. β-actin was used as loading control. Source data are provided as a Source Data file.

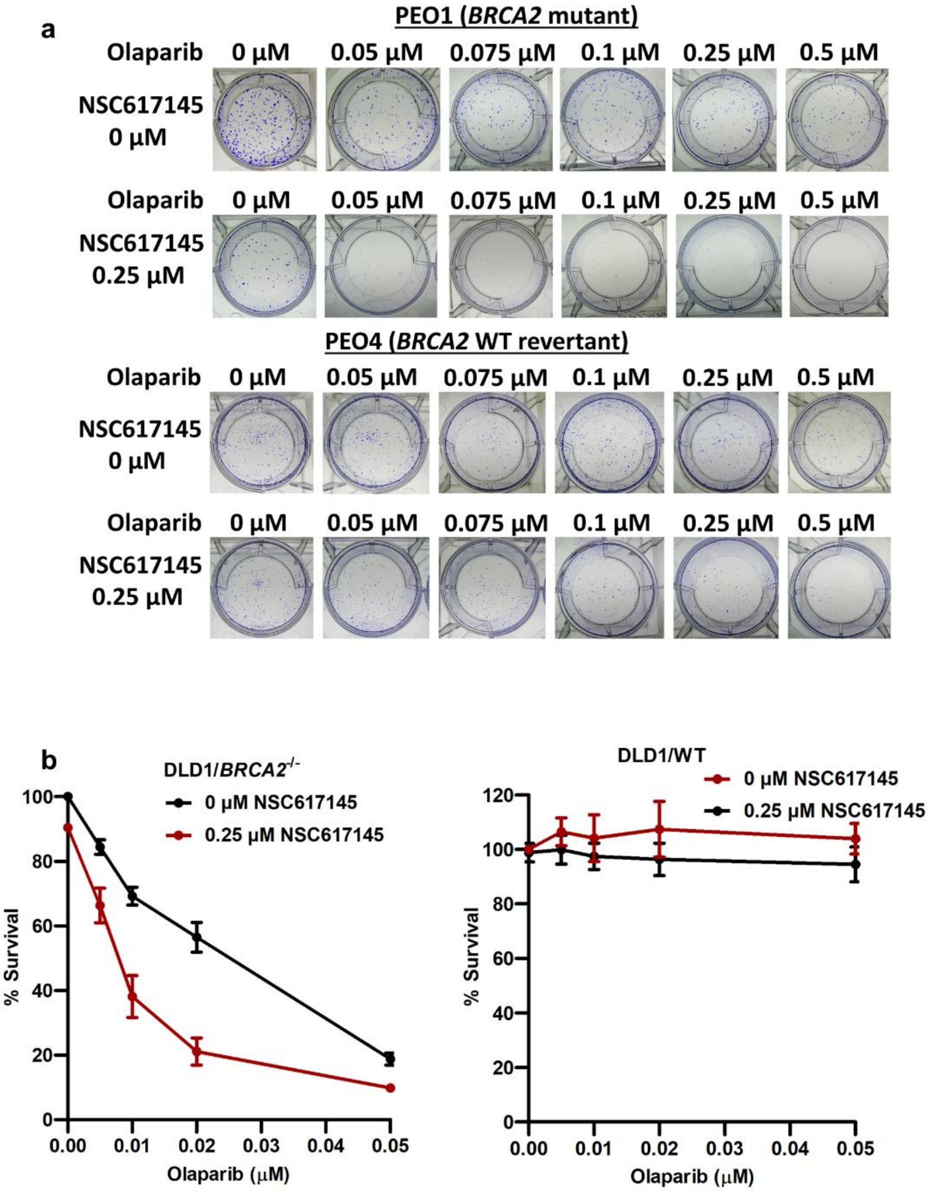

Supplementary Figure 14

Supplementary Figure 14. WRNi sensitizes *BRCA2*-deficient cancer cells to olaparib a, Representative images of colonies formed by PEO1 (upper panel) and PEO4 (lower panel) cells exposed to increasing concentrations of olaparib in presence or absence of NSC617145 (0.25 µM). **b**, Line graphs showing relative survival (%) of colonies formed by $BRCA2^{-/-}$ (left panel) and WT DLD1 cells treated with either indicated doses of olaparib only or in combination with 0.25 µM NSC617145. WT and $BRCA2^{-/-}$ DLD1 cells were plated on a 6-well plate at 1,000 cells/well and exposed to 0.25 µM NSC617145 and indicated doses of olaparib 4 h after cell seeding. Cells were allowed to form colonies in presence of the drugs for 12 days. Data represent mean ± SD of three (*n*=3) independent experiments. Source data are provided as a Source Data file.

Supplementary Table 1: Oligonucleotides used in the study

Oligo	Length	Sequence (5′ à3′)
A	80	CTT TAG CTG CAT ATT TAC AAC ATG TTG ACC TTC AGT A/iMe-isodC/A ATC TGC TCT GAT GCC GCA TAG TGT CAT GCC AGA GCT TTG TAC
B	81	CGG GTG TCG GGG CGC ATG ACA CTA TGC GGC ATC AGA GCA GAT TGT ACT GAA GGT CAA CAT GTT GTA AAT ATG CAG CTA AAG
C	43	GTA CAA AGC TCT GGC ATG ATA CTA TGC GGC ATC AGA GCA GAT T
D	50	TCA GTA CAA TCT GCT CTG ATG CCG CAT AGT ATC ATG CGC CCC GAC ACC CG
