# Supplementary material for: WRN helicase safeguards deprotected replication forks in BRCA2-mutated cancer cells
Source: Nat Commun. 2021 Nov 12;12:6561. doi: 10.1038/s41467-021-26811-w (PMC8590011; doi:10.1038/s41467-021-26811-w)
Supplement: Supplementary file 3 — Reporting Summary [file 41467_2021_26811_MOESM3_ESM.pdf]

Corresponding author(s): Robert M. Brosh Jr

Last updated by author(s): Oct 6, 2021

Reporting Summary

Nature Research wishes to improve the reproducibility of the work that we publish. This form provides structure for consistency and transparency in reporting. For further information on Nature Research policies, see our Editorial Policies and the Editorial Policy Checklist.

Statistics

For all statistical analyses, confirm that the following items are present in the figure legend, table legend, main text, or Methods section.

n/a	Confirmed	
☐	☒	The exact sample size (*n*) for each experimental group/condition, given as a discrete number and unit of measurement
☐	☒	A statement on whether measurements were taken from distinct samples or whether the same sample was measured repeatedly
☐	☒	The statistical test(s) used AND whether they are one- or two-sided<br>*Only common tests should be described solely by name; describe more complex techniques in the Methods section.*
☒	☐	A description of all covariates tested
☒	☐	A description of any assumptions or corrections, such as tests of normality and adjustment for multiple comparisons
☐	☒	A full description of the statistical parameters including central tendency (e.g. means) or other basic estimates (e.g. regression coefficient) AND variation (e.g. standard deviation) or associated estimates of uncertainty (e.g. confidence intervals)
☐	☒	For null hypothesis testing, the test statistic (e.g. *F*, *t*, *r*) with confidence intervals, effect sizes, degrees of freedom and *P* value noted<br>*Give P values as exact values whenever suitable.*
☒	☐	For Bayesian analysis, information on the choice of priors and Markov chain Monte Carlo settings
☒	☐	For hierarchical and complex designs, identification of the appropriate level for tests and full reporting of outcomes
☒	☐	Estimates of effect sizes (e.g. Cohen's *d*, Pearson's *r*), indicating how they were calculated

Our web collection on statistics for biologists contains articles on many of the points above.

Software and code

Policy information about availability of computer code

Data collection	DNA fibers, immunofluorescence and chromosome images were acquired using ZEN 3.0 (blue edition) software of Zeiss Axio Observer.Z1/7 microscope<br>Western blot chemiluminescent images were acquired using BioRad Chemidoc system<br>Radioactive gels were imaged using a Typhoon FLA 9500 (Cytiva)
Data analysis	DNA fibers, immunofluorescence and chromosomal aberrations were analyzed using ZEN 3.0 (blue edition) software of Zeiss<br>Immunohistochemistry data were analyzed using Image-J (Version 1.52s) (https://imagej.nih.gov/ij/)<br>Comet tail moment was analyzed using OpenComet (v1.3) analysis tool of ImageJ<br>Quantification of Western blots was done using Image lab 6.0.1 software (Biorad)<br>All statistical analyses were performed in GraphPad Prism, version 5.03<br>Line Graphs, scatter dot plots were generated in GraphPad Prism 5.03 or Microsoft Excel (Version 2108, Build 14326.20404). Bar graphs showing individual data points were generated in GraphPad Prism 8.3.1<br>Quantification of radiometric assays was done using ImageQuant TL (Cytiva)<br>Figure panels were assembled in Microsoft Powerpoint (Version 2108, Build 14326.20404)

For manuscripts utilizing custom algorithms or software that are central to the research but not yet described in published literature, software must be made available to editors and reviewers. We strongly encourage code deposition in a community repository (e.g. GitHub). See the Nature Research guidelines for submitting code & software for further information.

Data

Policy information about availability of data

All manuscripts must include a data availability statement. This statement should provide the following information, where applicable:

- Accession codes, unique identifiers, or web links for publicly available datasets
- A list of figures that have associated raw data
- A description of any restrictions on data availability

All data of the present study are available within the article and its supplementary information files or available from the corresponding author upon request. Source data are provided with this paper.

Field-specific reporting

Please select the one below that is the best fit for your research. If you are not sure, read the appropriate sections before making your selection.

☒ Life sciences ☐ Behavioural & social sciences ☐ Ecological, evolutionary & environmental sciences

For a reference copy of the document with all sections, see nature.com/documents/nr-reporting-summary-flat.pdf

Life sciences study design

All studies must disclose on these points even when the disclosure is negative.

Sample size	To determine sample size for mice xenograft experiment, resource equation method was used. No statistical analysis was computed to determine sample size for other experiments. Sample size was determined based on previous literature and previous experiments. For the DNA fiber experiments, 125-300 fibers were analyzed per experimental condition based on previously published literatures (Mijic et al, Nat Commun. 2017 Oct 16;8(1):859, PMID: 29038466; Ray Chaudhuri et al, Nature. 2016 Jul 21;535(7612):382-7, PMID: 27443740; Quinet et al, Methods Enzymol. 2017;591:55-82, PMID: 28645379) which is widely accepted in the field. Immunofluorescence and Comet assays (Awate et al, Nucleic Acids Res. 2020 Sep 18;48(16):9161-9180, PMID: 32797166; Thakar et al, Nat Commun. 2020 May 1;11(1):2147, PMID: 32358495); Mice xenograft immunohistochemistry (Wang et al; Cancer Res. 2016 May 1;76(9):2778-90, PMID: 27197267; Fok et al, Nat Commun. 2019 Nov 7;10(1):5065, PMID: 31699977); Western blots, Metaphase spread analysis (Aggarwal et al, Cancer Res. 2013 Sep 1;73(17):5497-507, PMID: 23867477); Biochemical assays (Aggarwal et al, Cancer Res. 2013 Sep 1;73(17):5497-507, PMID: 23867477; Sharma et al, Mol Biol Cell. 2004 Feb;15(2):734-50, PMID: 14657243); Colony survival and Cell viability assays (Lim et al; Mol Cell. 2018 Dec 20;72(6):925-941.e4, PMID: 30576655; Dungrawala et al, Mol Cell. 2017 Aug 3;67(3):374-386.e5, PMID: 28735897). Sample sizes are described in greater detail in the Figure Legends.
Data exclusions	No samples were excluded.
Replication	All cell-based and biochemical experiments were repeated independently at least two to three times with similar results. The numbers of independent biological replicates performed for each experiment is indicated in the Figure Legends. All results were reproducible.
Randomization	For mice xenograft experiment, mice were randomly allocated for each group and no selection criteria was used. For all cell-based assays, exponentially growing cells were counted, and equal number of cells were seeded (allocated) randomly in culture dishes or chamber slides for each experimental group set up for drug treatment, siRNA, or plasmid transfection. In biochemical assays, an equal volume of reaction cocktail mix containing reaction salts, water, and oligonucleotide substrates was distributed randomly in respective reaction tube prior to the addition of recombinant proteins.
Blinding	All measurements (data collection) and data analysis were done blindly for mice xenograft experiment. Blinding to group allocation during data collection was not relevant for Biochemical assays, Western blotting, DNA fiber experiments, immunofluorescence, comet assays, cell viability, and survival assays as researchers were aware of the respective experimental conditions.

Reporting for specific materials, systems and methods

We require information from authors about some types of materials, experimental systems and methods used in many studies. Here, indicate whether each material, system or method listed is relevant to your study. If you are not sure if a list item applies to your research, read the appropriate section before selecting a response.

Materials & experimental systems

n/a	Involved in the study
☐	☒ Antibodies
☐	☒ Eukaryotic cell lines
☒	☐ Palaeontology and archaeology
☐	☒ Animals and other organisms
☒	☐ Human research participants
☒	☐ Clinical data
☒	☐ Dual use research of concern

Methods

n/a	Involved in the study
☒	☐ ChIP-seq
☒	☐ Flow cytometry
☒	☐ MRI-based neuroimaging

Antibodies

Antibodies used

anti-WRN (4666, Mouse mAb (8H3), Cell Signaling, 1:1000),
anti-BRCA2 (A303-434A-M, Bethyl Laboratories, 1:1000; D9S6V, 10741, Cell Signaling, 1:1000),
anti-RAD51 (ab63801, Abcam, 1:1000),
anti-SMARCAL1 (A301-616A-T, Bethyl Laboratories, 1:1000),
anti-ZRANB3 (A303-033A-T, Bethyl Laboratories, 1:1000),
anti-HLTF (A300-230A-T, Bethyl Laboratories, 1:1000),
anti-β-actin (12620, D6A8, Cell Signaling, 1:5000),
anti-β-tubulin (N-20, sc-9935, Santa Cruz Biotechnology, Inc., 1:2000),
anti-DNA2 (ab96488, Abcam, 1:1000),
anti-Mus81 (MTA30 2G10/3, ab14387, Abcam, 1:1000),
anti-Rad52 (F-7, sc-365341, Santa Cruz Biotechnology Inc., 1:1000),
anti-Exonuclease 1 (ab95068, Abcam, 1:1000),
anti-MRE11 (12D7, ab214, Abcam, 1:1000),
anti-RECQL1 (H-110, sc-25547, Santa Cruz Biotechnology Inc., 1:1000),
anti-Topoisomerase I (556597, BD Pharmigen, 1:500)
anti-Lamin B1 (D9V6H, 13435, Cell Signaling, 1:1000)
anti-ORC2 (A302-734A, Bethyl Laboratories, 1:1000)
rat anti-CldU antibody (ab6326, Abcam, 1:200)
mouse anti-IdU antibody (347580, BD Pharmigen, 1:40)
anti-γ-H2AX (JBW301,05-636, Millipore Sigma, 1:1000 for WB and 1:100 for immunofluorescence)
anti-γ-H2AX (20E3, Rabbit mAb #9718, Cell Signaling, 1:100)
anti-DNA PKcs phospho-S2056 (EPR5670, ab124918, Abcam, 1:100)
anti-PCNA (PC10, sc-56, Santa Cruz Biotechnology Inc., 1:50)
anti-WRN (NB100-471, Novus Biologicals, 1:50)
anti-53BP1 (clone BP13, MAB3802, Millipore Sigma, 1:100)
anti-DNA PKcs phospho-T2609 (ab18356, Abcam, 1:100)
phospho-Histone H2AX (#16-193, Millipore, 1:100)
53BP1 (EPR2172(2), ab175933, Abcam, 1:200)
AlexaFluor 647-conjugated goat anti-mouse IgG (A-21235, ThermoFisher Scientific, 1:500)
AlexaFluor 488-conjugated goat anti-mouse IgG (A28175, ThermoFisher Scientific, 1:500)
AlexaFluor Plus 488 Goat anti-Rabbit IgG (A32731, ThermoFisher Scientific, 1:500)
AlexaFluor 647-conjugated anti-rat IgG (A-21247, ThermoFisher Scientific, 1:100)
AlexaFluor 488-conjugated anti-mouse IgG secondary antibody (A-11001, ThermoFisher Scientific, 1:100)
HRP-conjugated anti-mouse (32430, ThermoFisher Scientific, 1:2500)
HRP-conjugated anti-rabbit (656120, ThermoFisher Scientific, 1:2500)
HRP-conjugated anti-goat (SC-2020, Santa Cruz Biotechnology Inc., 1:2500)

Validation

Each primary antibody was validated for the specific applications by the manufacturers as follows:

anti-WRN (4666, Mouse mAb (8H3), Cell Signaling); validated by the manufacturer for application of WB, Species reactivity: Human, Mouse
anti-BRCA2 (A303-434A-M, Bethyl Laboratories; D9S6V, 10741, Cell Signaling); A303-434A-M, Bethyl Laboratories: validated by the manufacturer for application of WB, IP, Species reactivity: Human; D9S6V, 10741, Cell Signaling: validated by the manufacturer for application of WB, Species reactivity: Human
anti-β-tubulin (N-20, sc-9935, Santa Cruz Biotechnology, Inc.); validated by the manufacturer for application of WB, IP, IF, IHC, ELISA, Species reactivity: Human, Mouse and Rat
anti-RAD51 (ab63801, Abcam); validated by the manufacturer for application of WB, ICC/IF, Species reactivity: Human, Xenopus laevis
anti-SMARCAL1 (A301-616A-T, Bethyl Laboratories); validated by the manufacturer for application of WB, IP, Species reactivity: Human
anti-ZRANB3 (A303-033A-T, Bethyl Laboratories); validated by the manufacturer for application of WB, IP, Species reactivity: Human
anti-HLTF (A300-230A-T, Bethyl Laboratories); validated by the manufacturer for application of WB, IP, Species reactivity: Human,

Mouse

anti-β-actin (12620, D6A8, Cell Signaling); validated by the manufacturer for application of WB, Species reactivity: Human, Mouse, Rat, Monkey, D. melanogaster, Zebrafish

anti-DNA2 (ab96488, Abcam); validated by the manufacturer for application of WB, IHC-P, Species reactivity: Human

anti-Topoisomerase I (556597, BD Pharmigen); validated by the manufacturer for application of WB Species reactivity: Human

anti-Mus81 (MTA30 2G10/3, ab14387, Abcam); validated by the manufacturer for application of WB, Flow Cyt, Species reactivity: Human

anti-Rad52 (F-7, sc-365341, Santa Cruz Biotechnology Inc.); validated by the manufacturer for application of WB, IP, IF, IHC, ELISA, Species reactivity: Human, Mouse and Rat

anti-Exonuclease 1 (ab95068, Abcam); validated by the manufacturer for application of WB, IP, Species reactivity: Human

anti-MRE11 (12D7, ab214, Abcam); validated by the manufacturer for application of Flow Cyt, ICC/IF, WB, Species reactivity: Human and Rat

anti-RECQL1 (H-110, sc-25547, Santa Cruz Biotechnology Inc.); validated by the manufacturer for application of WB, IP, IF, IHC, ELISA, Species reactivity: Human, Mouse and Rat

anti-Lamin B1 (D9V6H, 13435, Cell Signaling); validated by the manufacturer for application of WB, IP, Species reactivity: Human, Mouse and Rat

anti-ORC2 (A302-734A, Bethyl Laboratories); validated by the manufacturer for application of WB, IP, IHC, Species reactivity: Human

rat anti-CldU antibody (ab6326, Abcam); validated by the manufacturer for application of ICC/IF, IHC-P, Flow Cyt, Species reactivity: Species independent, widely used antibody to detect CldU-labeled DNA fibers in DNA Fiber experiments (Quinet et al, Methods Enzymol. 2017;591:55-82, PMID: 28645379)

mouse anti-IdU antibody (347580, BD Pharmigen); validated by the manufacturer for application of Flow Cyt, Species reactivity: Not applicable, widely used antibody to detect IdU-labeled DNA fibers in DNA Fiber experiments (Quinet et al, Methods Enzymol. 2017;591:55-82, PMID: 28645379)

anti-γ-H2AX (JBW301,05-636, Millipore Sigma); validated by the manufacturer for application of ChIP, ICC, IF, WB, Species reactivity: Vertebrates

anti-γ-H2AX (20E3, Rabbit mAb #9718, Cell Signaling); validated by the manufacturer for application of WB, IHC-P, IF-IC, F, Species reactivity: Human, Mouse, Rat, Monkey

anti-DNA PKcs phospho-S2056 (EPR5670, ab124918, Abcam); validated by the manufacturer for application of ICC/IF, WB, IHC-P, ELISA, Species reactivity: Human

anti-PCNA (PC10, sc-56, Santa Cruz Biotechnology Inc.); validated by the manufacturer for application of WB, IP, IF, IHC, and Flow cytometry, Species reactivity: mouse, rat, human, insect and S. pombe

anti-WRN (NB100-471, Novus Biologicals); validated by the manufacturer for application of WB, ICC/IF, IHC, IP, ChIP, Species reactivity: Human

anti-53BP1 (clone BP13, MAB3802, Millipore Sigma); validated by the manufacturer for application of WB, IF, Species reactivity: Human

anti-DNA PKcs phospho-T2609 (ab18356, Abcam); validated by the manufacturer for application of ICC/IF, WB, Species reactivity: Human

phospho-Histone H2AX (#16-193, Millipore); validated by the manufacturer for application of ICC, WB, Species reactivity: Human

53BP1 (EPR2172(2), ab175933, Abcam); validated by the manufacturer for application of WB, IHC-P, ICC/IF, Flow Cyt, Knockout validated, Species reactivity: Mouse, Rat, Human

anti-WRN (4666, Mouse mAb (8H3), Cell Signaling); validated by the manufacturer for application of WB, Species reactivity: Human, Mouse

anti-BRCA2 (A303-434A-M, Bethyl Laboratories; D9S6V, 10741, Cell Signaling); A303-434A-M, Bethyl Laboratories: validated by the manufacturer for application of WB, IP, Species reactivity: Human; D9S6V, 10741, Cell Signaling: validated by the manufacturer for application of WB, Species reactivity: Human

anti-β-tubulin (N-20, sc-9935, Santa Cruz Biotechnology, Inc.); validated by the manufacturer for application of WB, IP, IF, IHC, ELISA, Species reactivity: Human, Mouse and Rat

anti-RAD51 (ab63801, Abcam); validated by the manufacturer for application of WB, ICC/IF, Species reactivity: Human, Xenopus laevis

anti-SMARCAL1 (A301-616A-T, Bethyl Laboratories); validated by the manufacturer for application of WB, IP, Species reactivity: Human

anti-ZRANB3 (A303-033A-T, Bethyl Laboratories); validated by the manufacturer for application of WB, IP, Species reactivity: Human

anti-HLTF (A300-230A-T, Bethyl Laboratories); validated by the manufacturer for application of WB, IP, Species reactivity: Human, Mouse

anti-β-actin (12620, D6A8, Cell Signaling); validated by the manufacturer for application of WB, Species reactivity: Human, Mouse, Rat, Monkey, D. melanogaster, Zebrafish

anti-DNA2 (ab96488, Abcam); validated by the manufacturer for application of WB, IHC-P, Species reactivity: Human

anti-Topoisomerase I (556597, BD Pharmigen); validated by the manufacturer for application of WB Species reactivity: Human

anti-Mus81 (MTA30 2G10/3, ab14387, Abcam); validated by the manufacturer for application of WB, Flow Cyt, Species reactivity: Human

anti-Rad52 (F-7, sc-365341, Santa Cruz Biotechnology Inc.); validated by the manufacturer for application of WB, IP, IF, IHC, ELISA, Species reactivity: Human, Mouse and Rat

anti-Exonuclease 1 (ab95068, Abcam); validated by the manufacturer for application of WB, IP, Species reactivity: Human

anti-MRE11 (12D7, ab214, Abcam); validated by the manufacturer for application of Flow Cyt, ICC/IF, WB, Species reactivity: Human and Rat

anti-RECQL1 (H-110, sc-25547, Santa Cruz Biotechnology Inc.); validated by the manufacturer for application of WB, IP, IF, IHC, ELISA, Species reactivity: Human, Mouse and Rat

anti-Lamin B1 (D9V6H, 13435, Cell Signaling); validated by the manufacturer for application of WB, IP, Species reactivity: Human, Mouse and Rat

anti-ORC2 (A302-734A, Bethyl Laboratories); validated by the manufacturer for application of WB, IP, IHC, Species reactivity: Human

rat anti-CldU antibody (ab6326, Abcam); validated by the manufacturer for application of ICC/IF, IHC-P, Flow Cyt, Species reactivity: Species independent, widely used antibody to detect CldU-labeled DNA fibers in DNA Fiber experiments (Quinet et al, Methods Enzymol. 2017;591:55-82, PMID: 28645379)

mouse anti-IdU antibody (347580, BD Pharmigen); validated by the manufacturer for application of Flow Cyt, Species reactivity: Not applicable, widely used antibody to detect IdU-labeled DNA fibers in DNA Fiber experiments (Quinet et al, Methods Enzymol. 2017;591:55-82, PMID: 28645379)
anti-γ-H2AX (JBW301,05-636, Millipore Sigma); validated by the manufacturer for application of ChIP, ICC, IF, WB, Species reactivity: Vertebrates
anti-γ-H2AX (20E3, Rabbit mAb #9718, Cell Signaling); validated by the manufacturer for application of WB, IHC-P, IF-IC, F, Species reactivity: Human, Mouse, Rat, Monkey
anti-DNA PKcs phospho-S2056 (EPR5670, ab124918, Abcam); validated by the manufacturer for application of ICC/IF, WB, IHC-P, ELISA, Species reactivity: Human
anti-PCNA (PC10, sc-56, Santa Cruz Biotechnology Inc.); validated by the manufacturer for application of WB, IP, IF, IHC, and Flow cytometry, Species reactivity: mouse, rat, human, insect and S. pombe
anti-WRN (NB100-471, Novus Biologicals); validated by the manufacturer for application of WB, ICC/IF, IHC, IP, ChIP, Species reactivity: Human
anti-53BP1 (clone BP13, MAB3802, Millipore Sigma); validated by the manufacturer for application of WB, IF, Species reactivity: Human
anti-DNA PKcs phospho-T2609 (ab18356, Abcam); validated by the manufacturer for application of ICC/IF, WB, Species reactivity: Human
phospho-Histone H2AX (#16-193, Millipore); validated by the manufacturer for application of ICC, WB, Species reactivity: Human
53BP1 (EPR2172(2), ab175933, Abcam); validated by the manufacturer for application of WB, IHC-P, ICC/IF, Flow Cyt, Knockout validated, Species reactivity: Mouse, Rat, Human

Eukaryotic cell lines

Policy information about cell lines

Cell line source(s)	DLD1 WT and BRCA2-/- (Horizon Discovery)<br>PEO1 and PEO4 cells, gift from Dr. Lee Zou (Massachusetts General Hospital Cancer Center, Harvard Medical School). PEO1 and PEO4 cell lines were originally derived from the ascites of a patient with ovarian serous adenocarcinoma (Langdon et al, Cancer Res. 1988 Nov 1;48(21):6166-72, PMID: 3167863; Wolf et al, Int J Cancer. 1987 Jun 15;39(6):695-702, PMID: 3583449)<br>SKOV-3, MDA-MB-231, U2OS and HeLa cells (ATCC)<br>FANCD2-/- PD20 cells and corrected counterparts were obtained Fanconi Anemia Cell Repository at Oregon Health & Science University (Portland, OR)<br>U2OS/WRN-/-, DLD1/WRN-/-, HeLa/RECQL1-/- cells were generated in this study
Authentication	DLD1/WT and CRISPR DLD1/BRCA2-/- cell lines cell lines were authenticated by Horizon Discovery using PCR amplification and Sanger Sequencing to confirm the mutation at the genomic level. SKOV-3, MDA-MB-231, U2OS and HeLa cells were authenticated by ATCC using STR profiling. PEO1 and PEO4 cells were authenticated by BRCA2 sequencing (Sakai et al, Cancer Res. 2009 Aug 15;69(16):6381-6, PMID: 19654294). U2OS/WRN-/-, DLD1/WRN-/-, HeLa/RECQL1-/-, and FANCD2-/- PD20 cells were validated based on expression of the respective CRISPR target or mutated genes.
Mycoplasma contamination	All cell lines were tested negative for mycoplasma contamination.
Commonly misidentified lines (See ICLAC register)	No misidentified cell line was used

Animals and other organisms

Policy information about studies involving animals; ARRIVE guidelines recommended for reporting animal research

Laboratory animals	Athymic nude mice (C3H/HeNCr-nu), females, 8 wk. old
Wild animals	No wild animals were used in the study
Field-collected samples	No field collected samples were used in the study
Ethics oversight	Mice were maintained as outlined in the Guide for the Care and Use of Laboratory Animals, under a protocol approved by the Animal Care and Use Committee (ACUC) of NCI-Frederick.

Note that full information on the approval of the study protocol must also be provided in the manuscript.
