# Supplementary material for: WRN helicase safeguards deprotected replication forks in BRCA2-mutated cancer cells
Source: Nat Commun. 2021 Nov 12;12:6561. doi: 10.1038/s41467-021-26811-w (PMC8590011; doi:10.1038/s41467-021-26811-w)
Supplement: Supplementary file 4 — Source Data [file 41467_2021_26811_MOESM4_ESM.zip › Brosh Source Data/286547_2_supp_5954041_r0k7kh.pptx]

## Slide 1
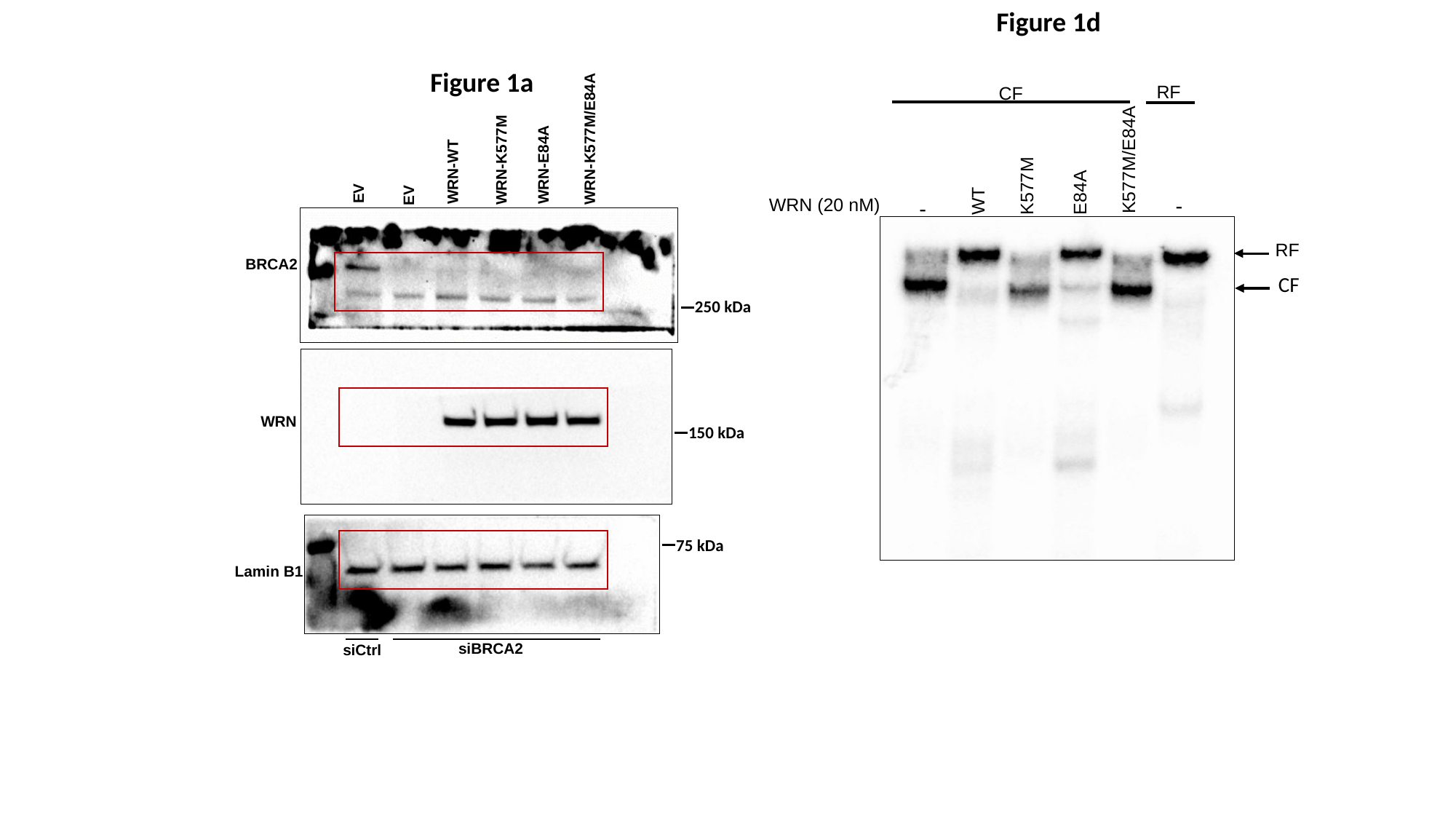

Figure 1d
Figure 1a
BRCA2
WRN
Lamin B1
RF
CF
WRN-K577M/E84A
K577M/E84A
WRN-K577M
WRN-E84A
WRN-WT
K577M
E84A
EV
EV
WT
-
WRN (20 nM)
-
RF
CF
250 kDa
150 kDa
75 kDa
siBRCA2
siCtrl

## Slide 2
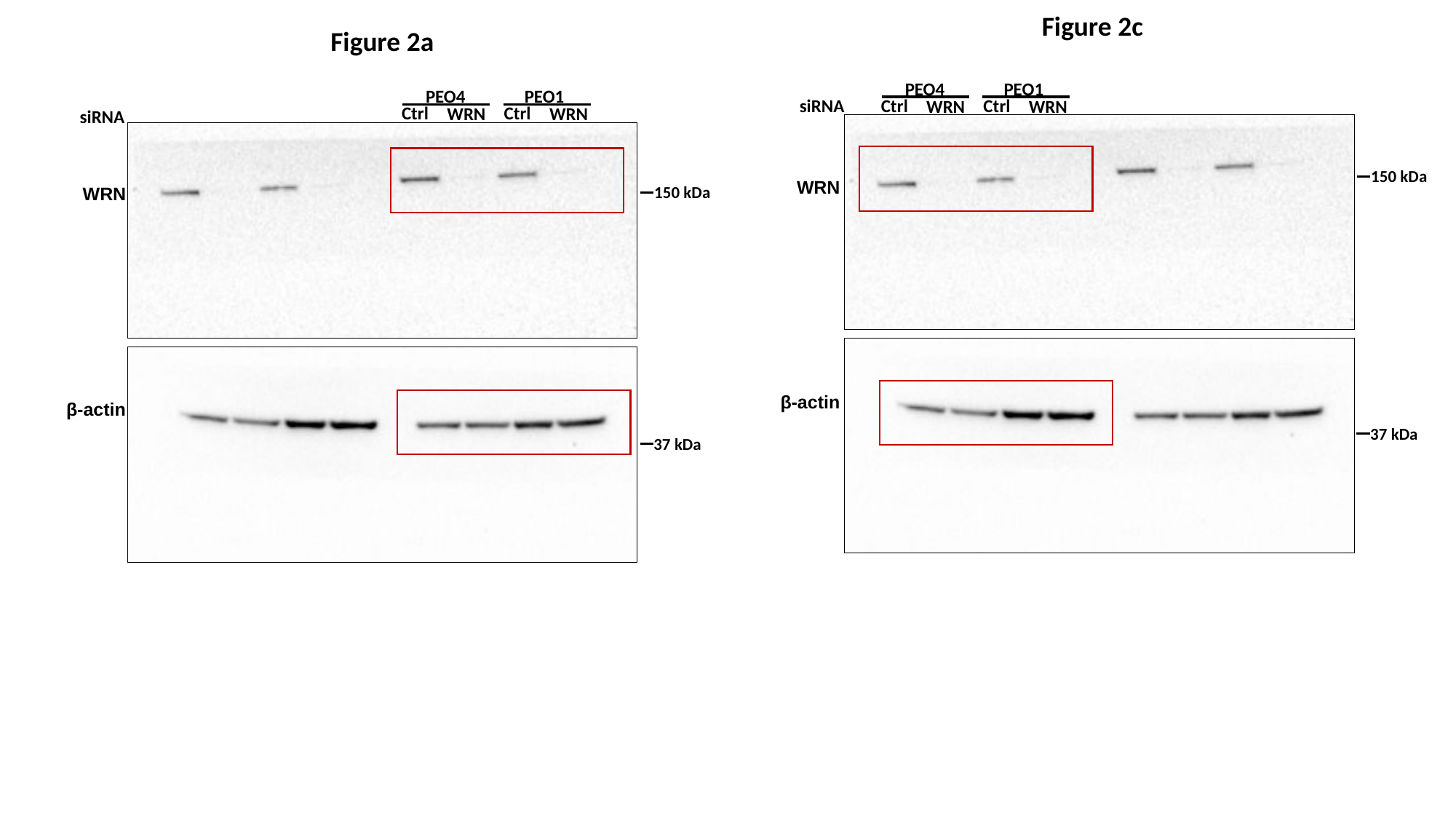

Figure 2c
Figure 2a
PEO4
PEO1
PEO4
PEO1
Ctrl
Ctrl
siRNA
WRN
WRN
Ctrl
Ctrl
WRN
WRN
siRNA
150 kDa
WRN
150 kDa
WRN
β-actin
β-actin
37 kDa
37 kDa

## Slide 3
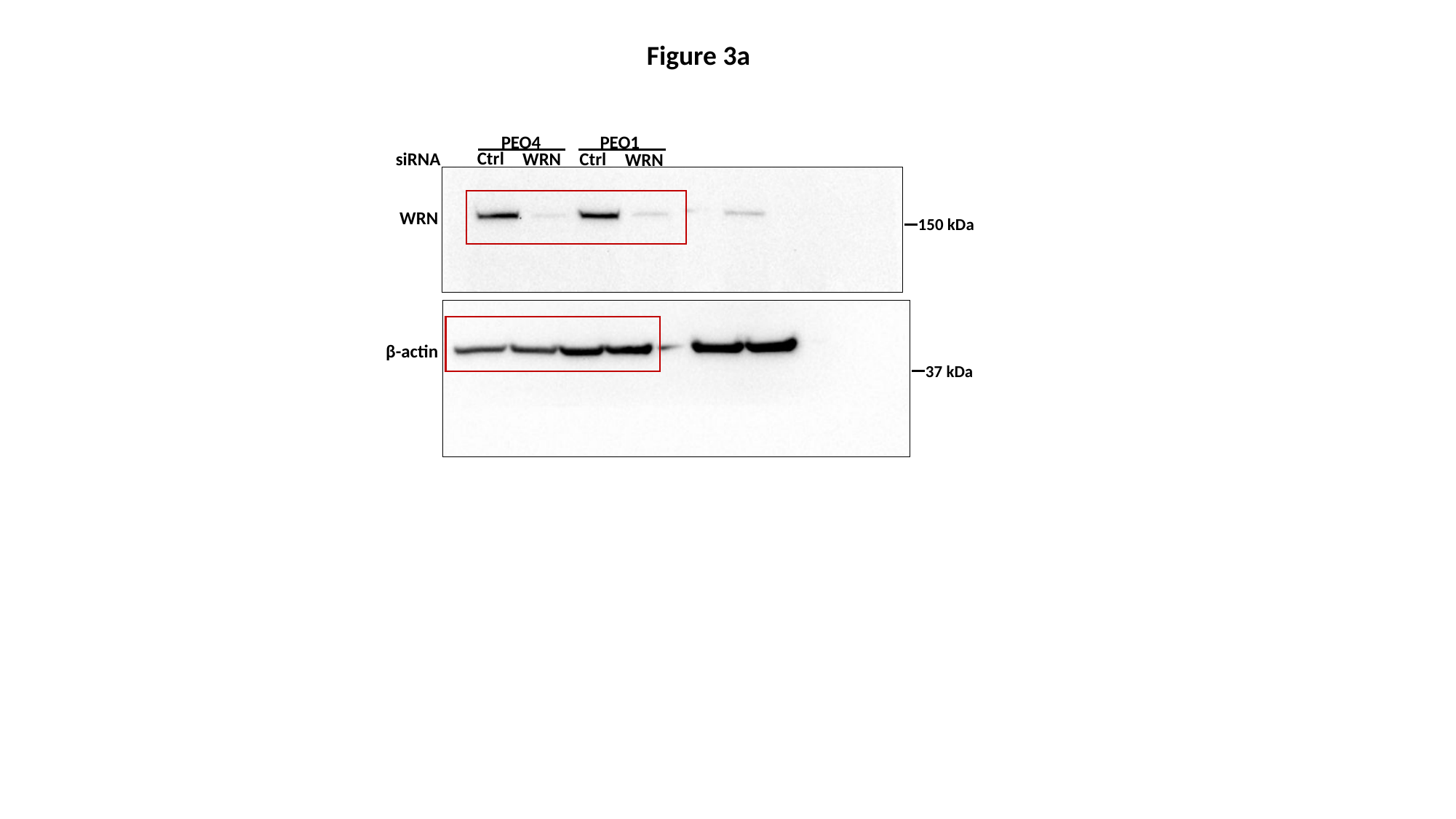

Figure 3a
PEO4
PEO1
Ctrl
Ctrl
siRNA
WRN
WRN
WRN
150 kDa
β-actin
37 kDa

## Slide 4
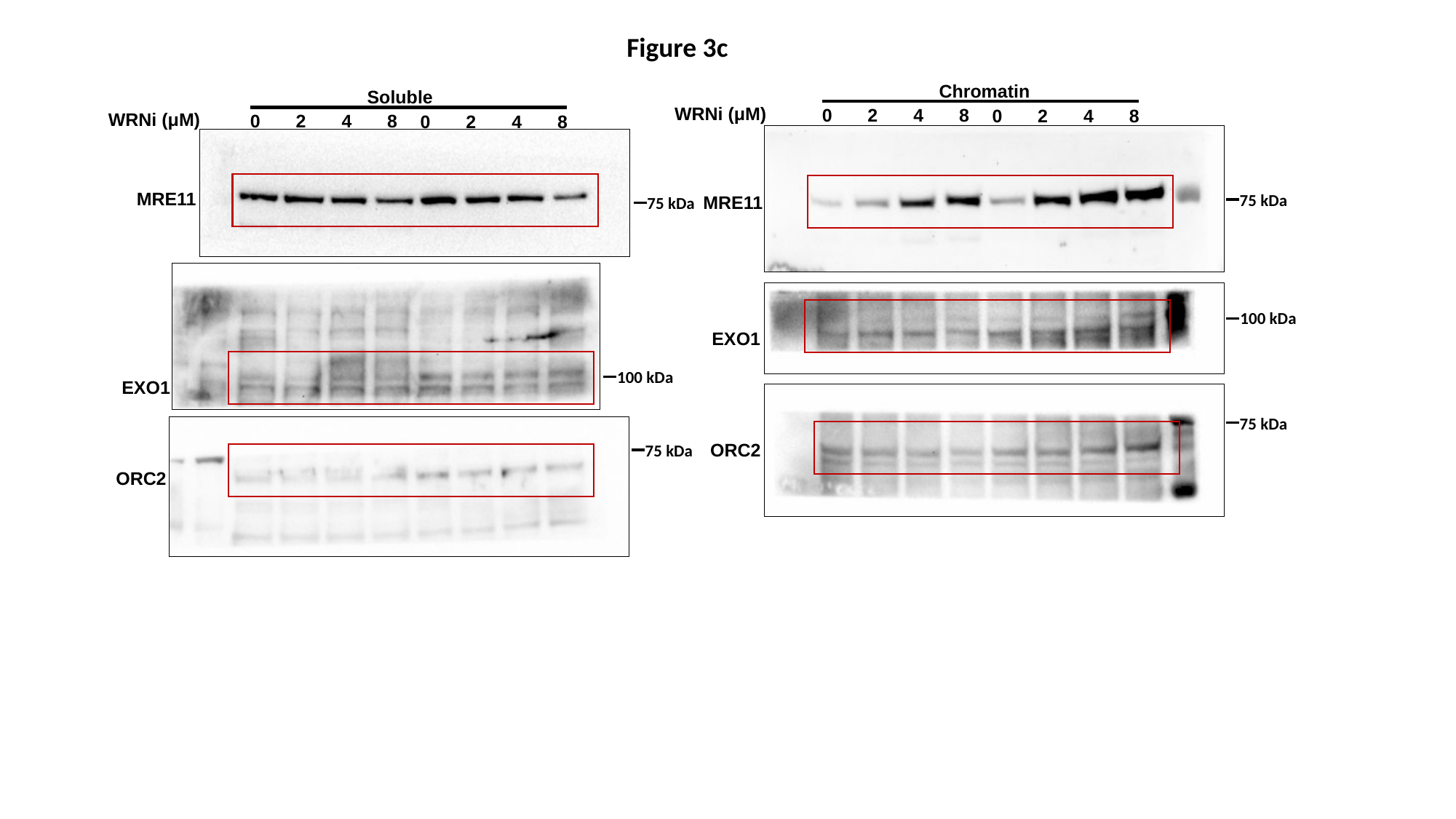

Figure 3c
Chromatin
Soluble
WRNi (μM)
0 2 4 8
0 2 4 8
WRNi (μM)
0 2 4 8
0 2 4 8
MRE11
75 kDa
MRE11
75 kDa
100 kDa
EXO1
100 kDa
EXO1
75 kDa
ORC2
75 kDa
ORC2

## Slide 5
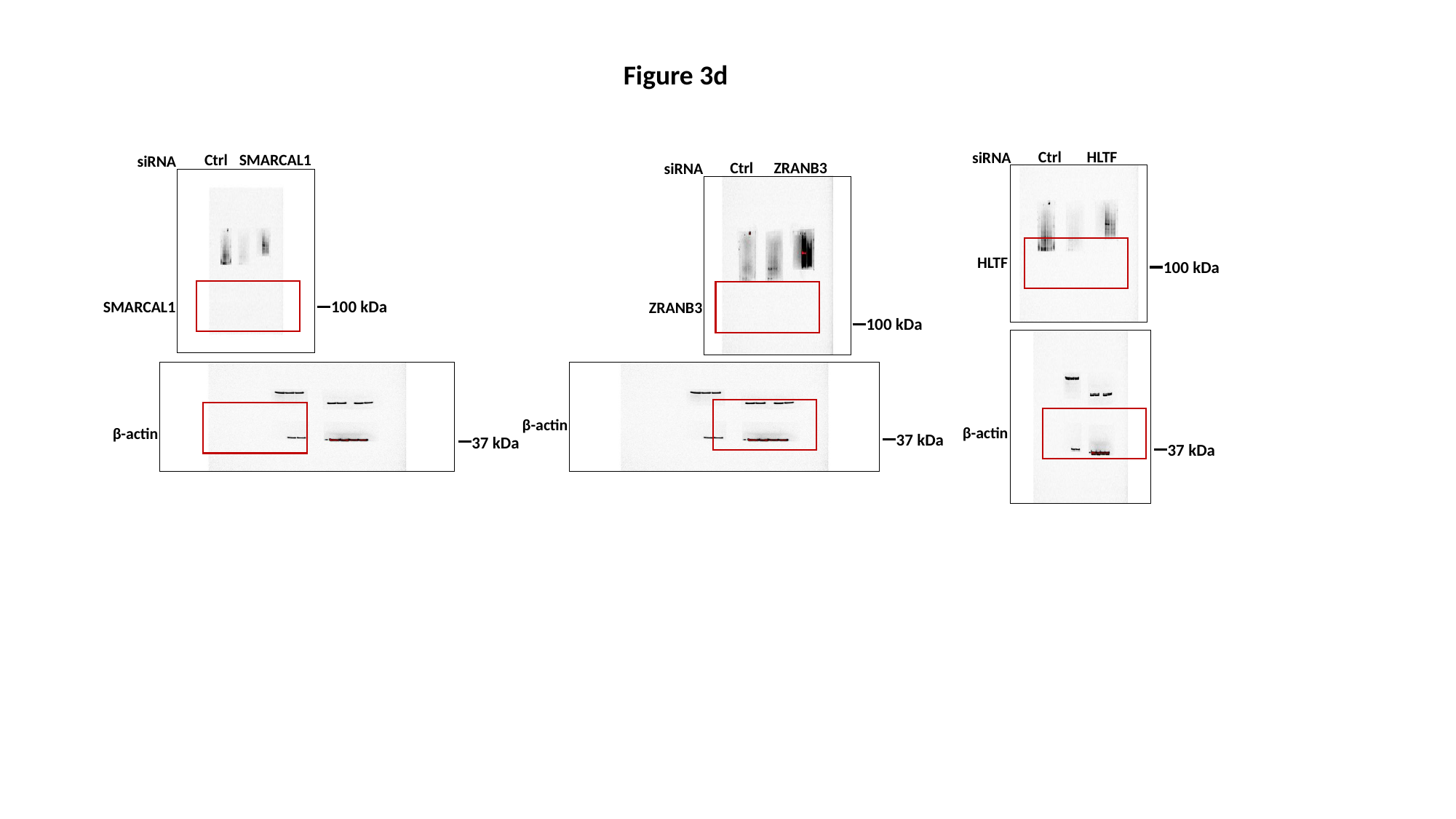

Figure 3d
HLTF
Ctrl
siRNA
SMARCAL1
Ctrl
siRNA
ZRANB3
Ctrl
siRNA
HLTF
100 kDa
100 kDa
SMARCAL1
ZRANB3
100 kDa
β-actin
β-actin
β-actin
37 kDa
37 kDa
37 kDa

## Slide 6
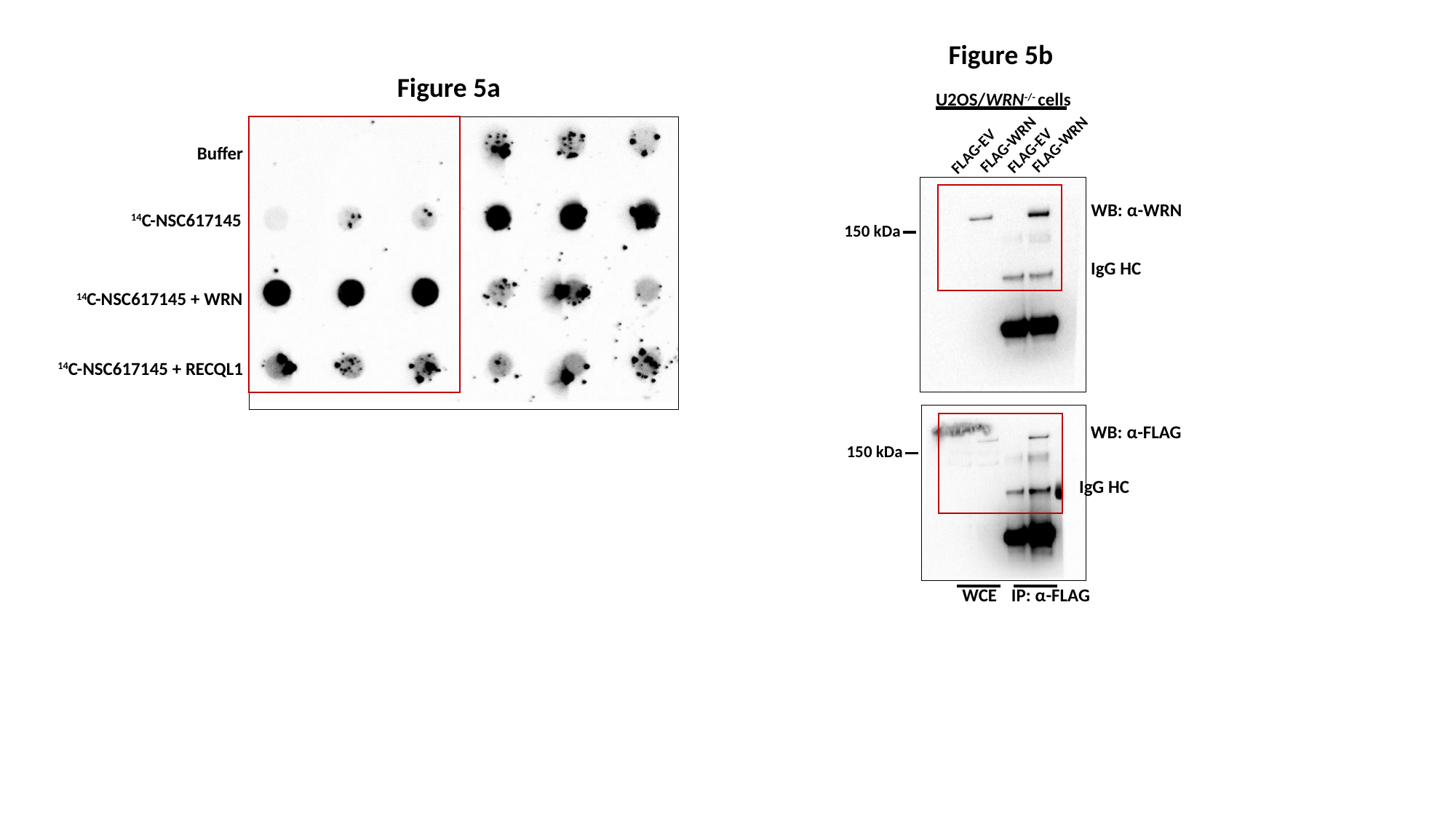

Figure 5b
Figure 5a
U2OS/WRN-/- cells
Buffer
14C-NSC617145
14C-NSC617145 + WRN
14C-NSC617145 + RECQL1
FLAG-WRN
FLAG-WRN
FLAG-EV
FLAG-EV
WB: α-WRN
150 kDa
IgG HC
WB: α-FLAG
150 kDa
IgG HC
WCE
IP: α-FLAG

## Slide 7
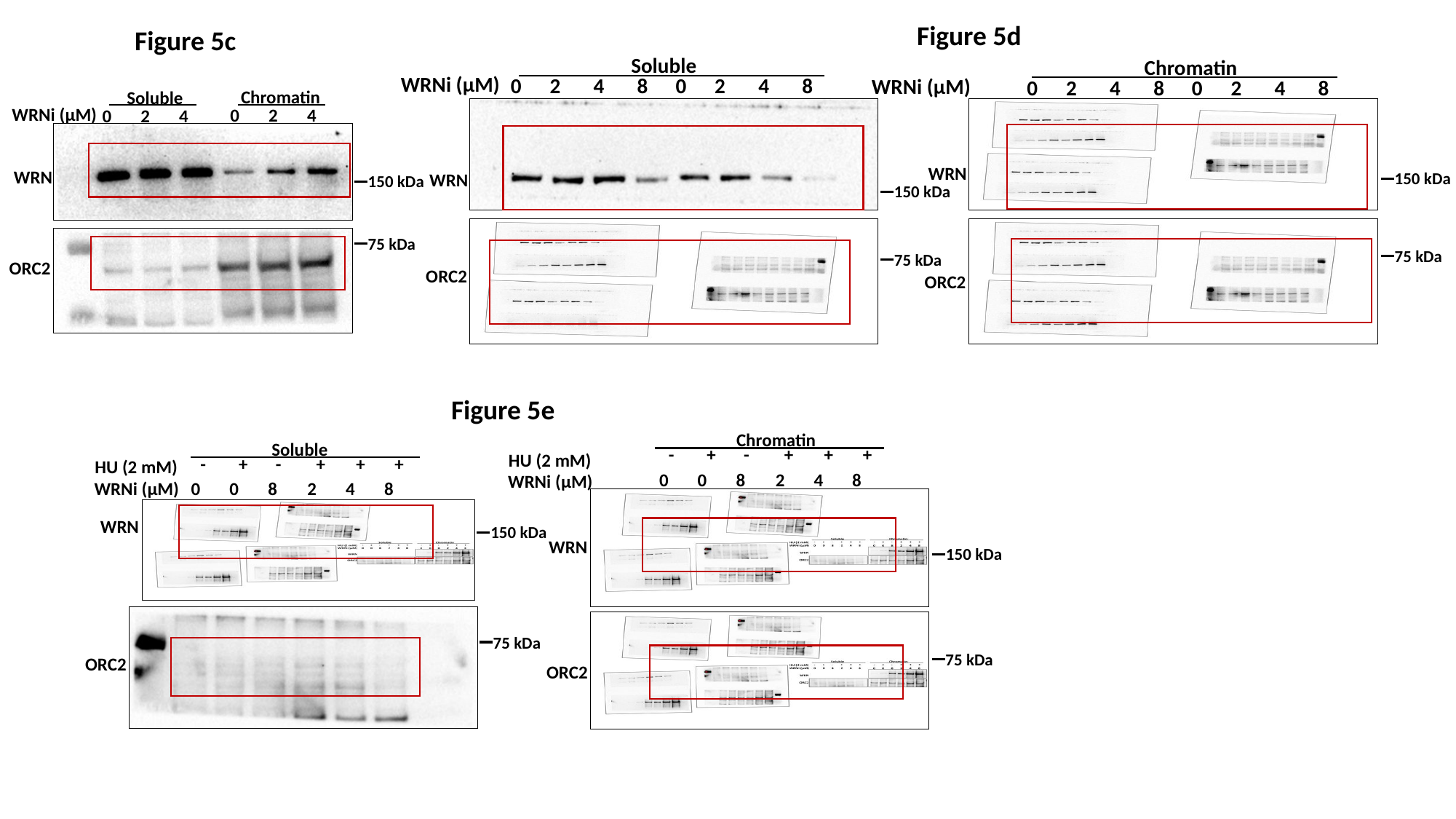

Figure 5d
WRN
ORC2
Figure 5c
WRN
ORC2
Soluble
Chromatin
WRNi (μM)
0 2 4 8
0 2 4 8
WRNi (μM)
0 2 4 8
0 2 4 8
Chromatin
Soluble
WRNi (μM)
0 2 4
0 2 4
WRN
150 kDa
150 kDa
150 kDa
75 kDa
75 kDa
75 kDa
ORC2
Figure 5e
WRN
ORC2
Chromatin
Soluble
+
+
+
-
-
+
HU (2 mM)
+
+
+
-
-
+
HU (2 mM)
2 4 8
0 0 8
WRNi (μM)
WRNi (μM)
2 4 8
0 0 8
150 kDa
WRN
150 kDa
75 kDa
75 kDa
ORC2

## Slide 8
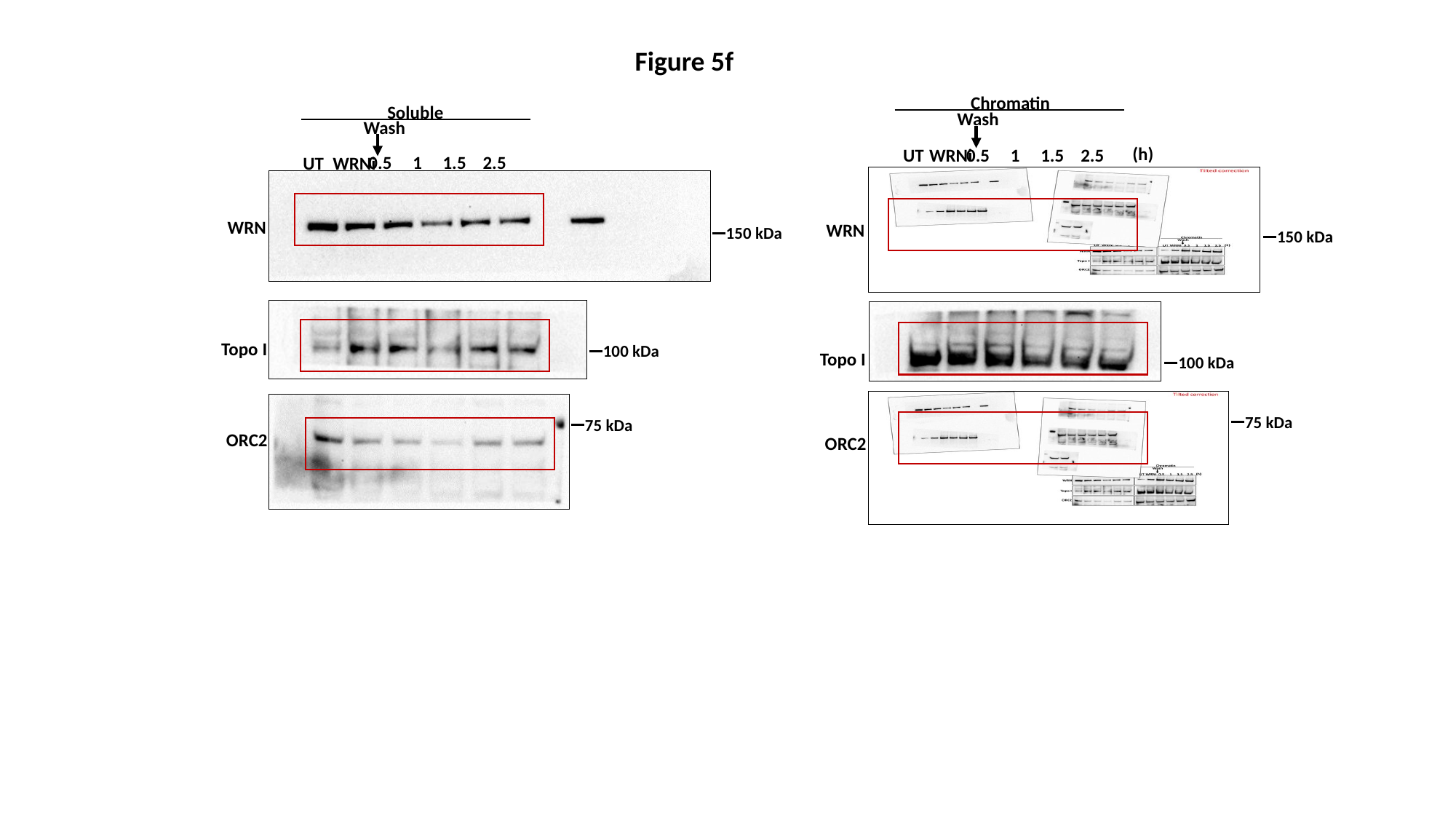

Figure 5f
Chromatin
Soluble
Wash
Wash
(h)
WRNi
 0.5 1 1.5 2.5
UT
 0.5 1 1.5 2.5
WRNi
UT
WRN
WRN
150 kDa
150 kDa
Topo I
100 kDa
Topo I
100 kDa
75 kDa
75 kDa
ORC2
ORC2

## Slide 9
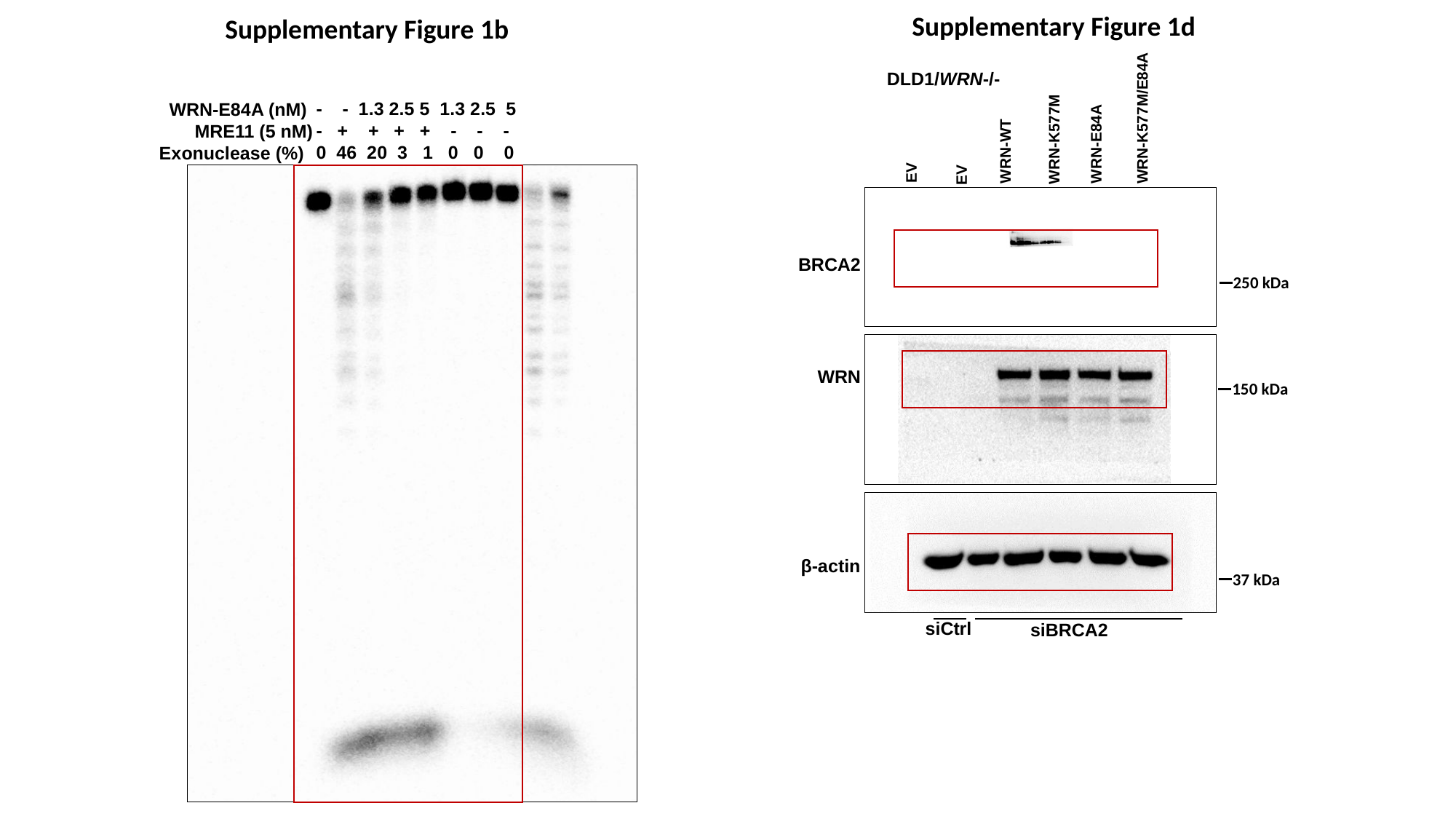

Supplementary Figure 1d
Supplementary Figure 1b
DLD1/WRN-/-
- - 1.3 2.5 5 1.3 2.5 5
- + + + + - - -
0 46 20 3 1 0 0 0
 WRN-E84A (nM)
 MRE11 (5 nM)
Exonuclease (%)
WRN-K577M/E84A
WRN-K577M
WRN-E84A
WRN-WT
EV
EV
BRCA2
250 kDa
WRN
150 kDa
β-actin
37 kDa
siCtrl
siBRCA2

## Slide 10
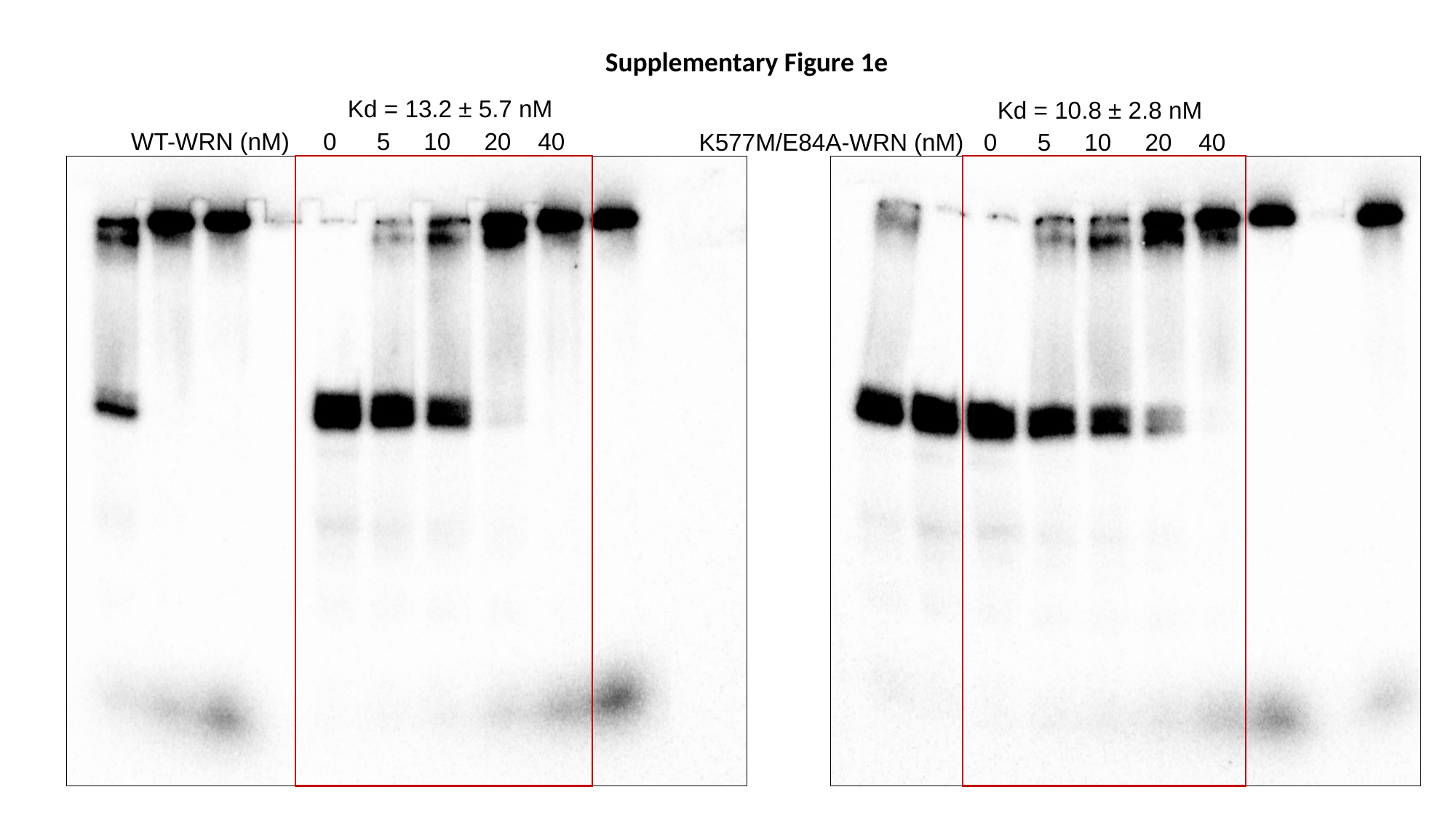

Supplementary Figure 1e
Kd = 13.2 ± 5.7 nM
Kd = 10.8 ± 2.8 nM
WT-WRN (nM) 0 5 10 20 40
K577M/E84A-WRN (nM) 0 5 10 20 40

## Slide 11
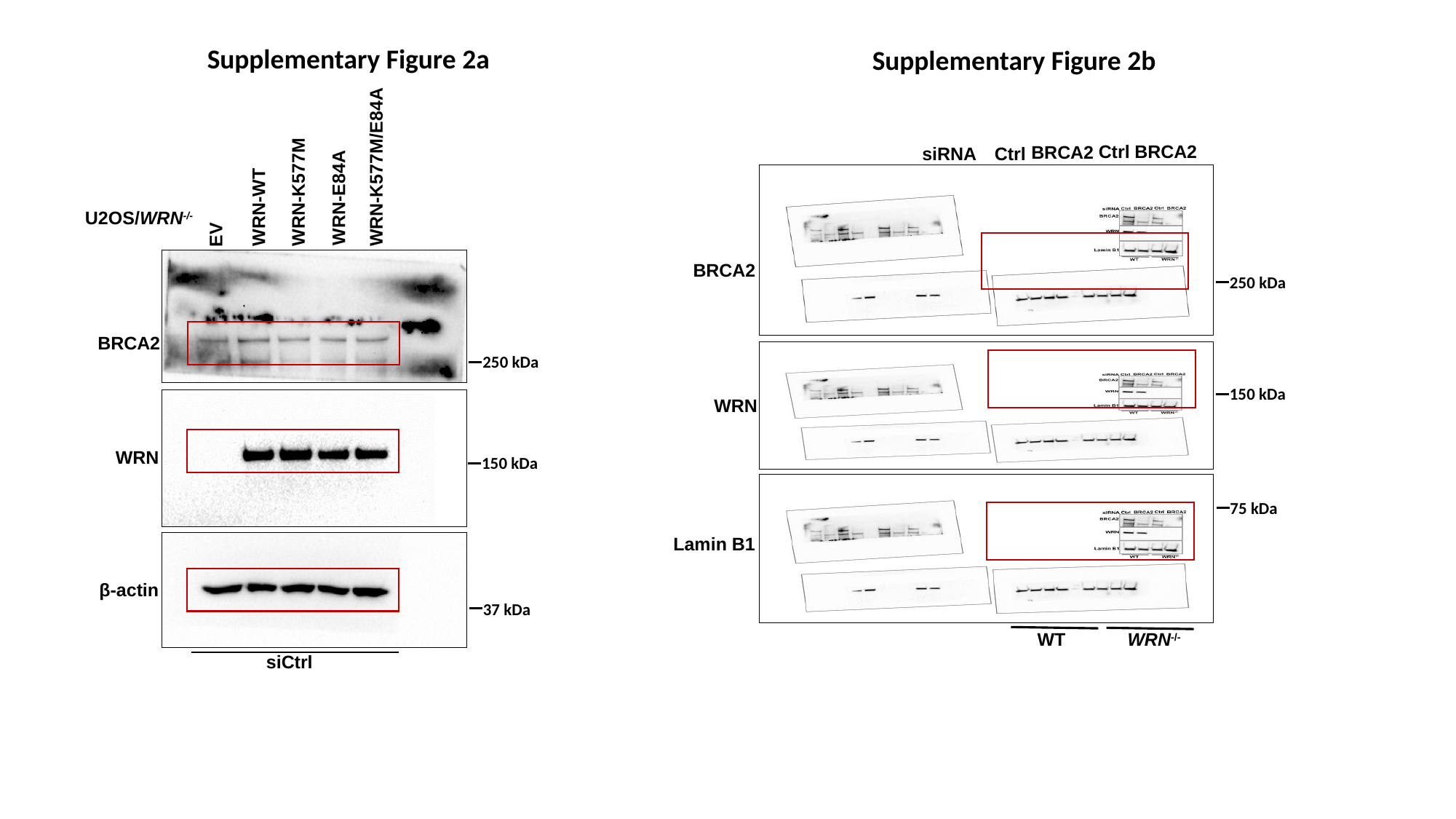

Supplementary Figure 2a
Supplementary Figure 2b
Ctrl
BRCA2
BRCA2
siRNA
Ctrl
WRN-K577M/E84A
BRCA2
WRN
Lamin B1
WRN-K577M
WRN-E84A
WRN-WT
 U2OS/WRN-/-
EV
BRCA2
WRN
β-actin
250 kDa
250 kDa
150 kDa
150 kDa
75 kDa
37 kDa
WT
WRN-/-
siCtrl

## Slide 12
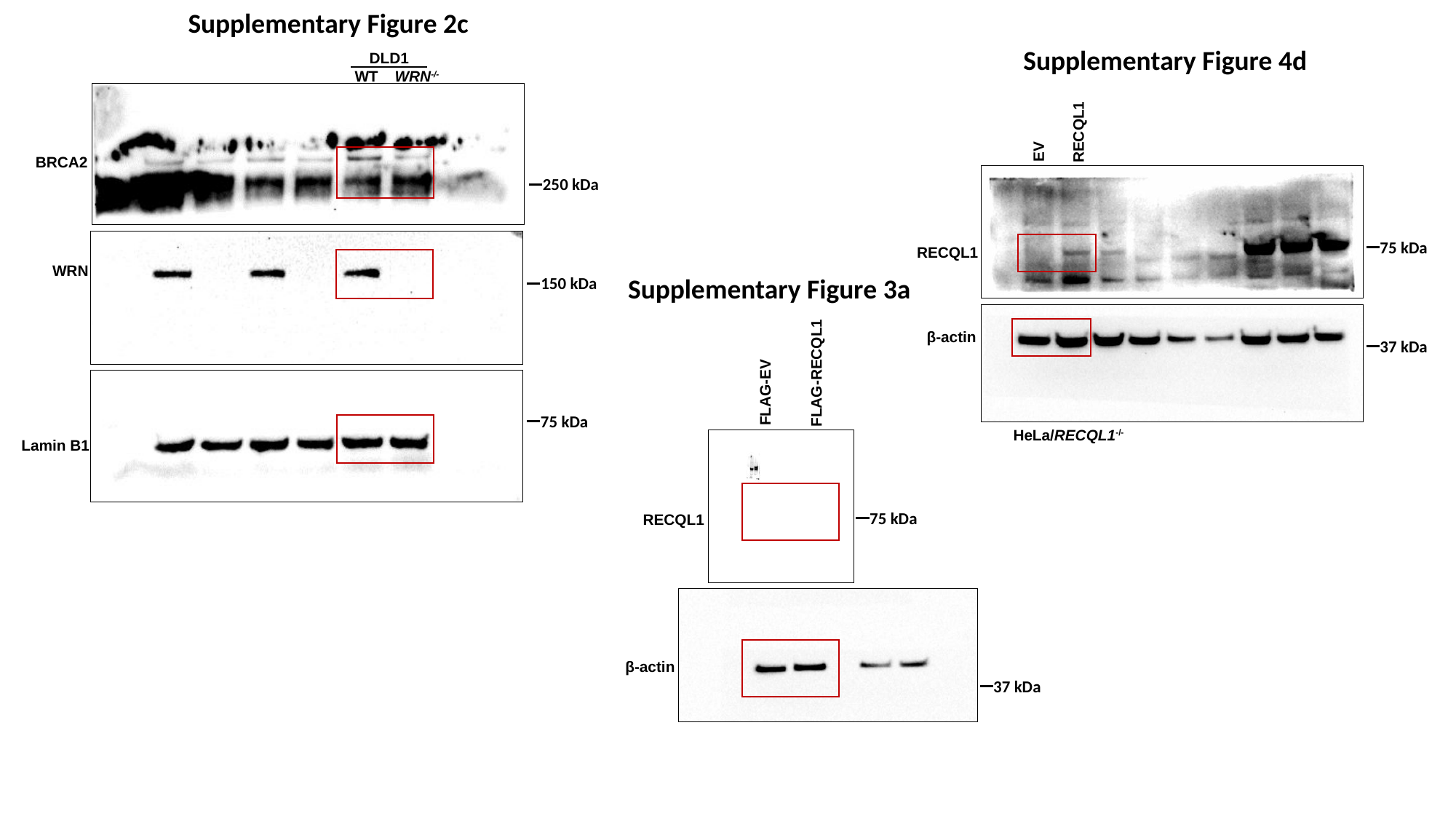

Supplementary Figure 2c
Supplementary Figure 4d
DLD1
WT
WRN-/-
BRCA2
WRN
Lamin B1
250 kDa
150 kDa
75 kDa
RECQL1
EV
RECQL1
β-actin
75 kDa
Supplementary Figure 3a
37 kDa
FLAG-RECQL1
FLAG-EV
HeLa/RECQL1-/-
RECQL1
β-actin
75 kDa
37 kDa

## Slide 13
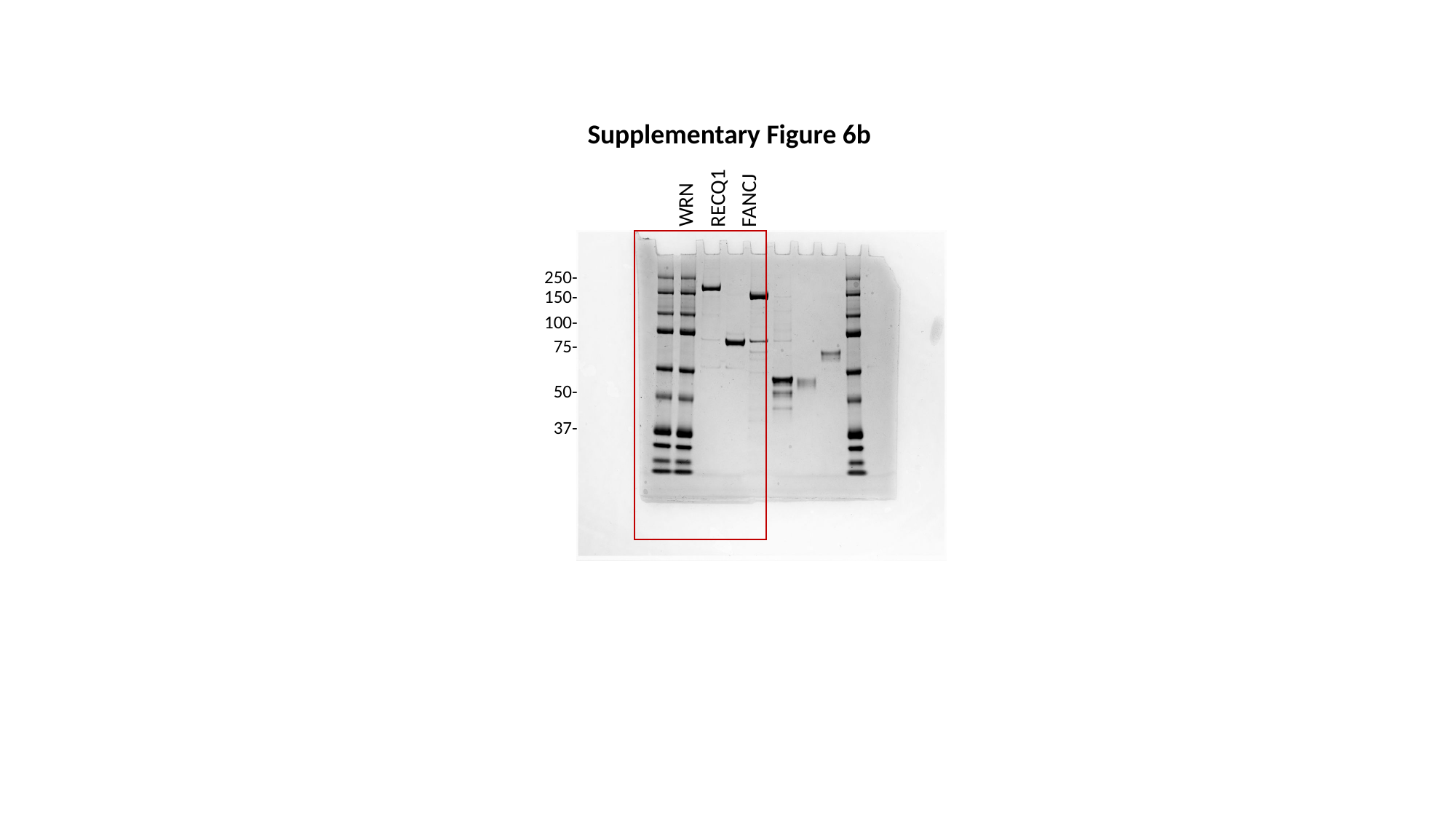

Supplementary Figure 6b
RECQ1
FANCJ
WRN
250-
150-
100-
75-
50-
37-

## Slide 14
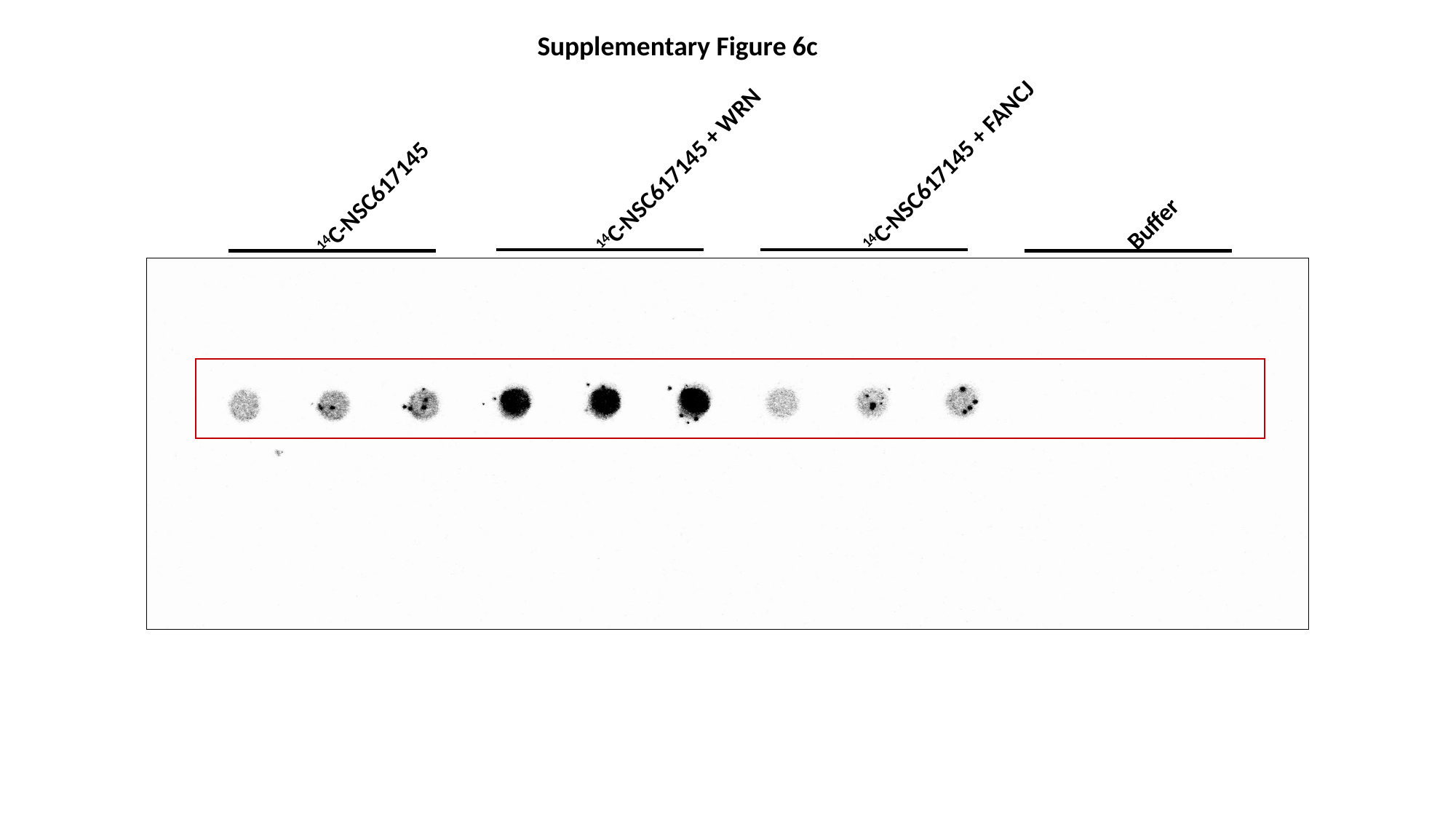

Supplementary Figure 6c
14C-NSC617145 + FANCJ
14C-NSC617145 + WRN
14C-NSC617145
Buffer

## Slide 15
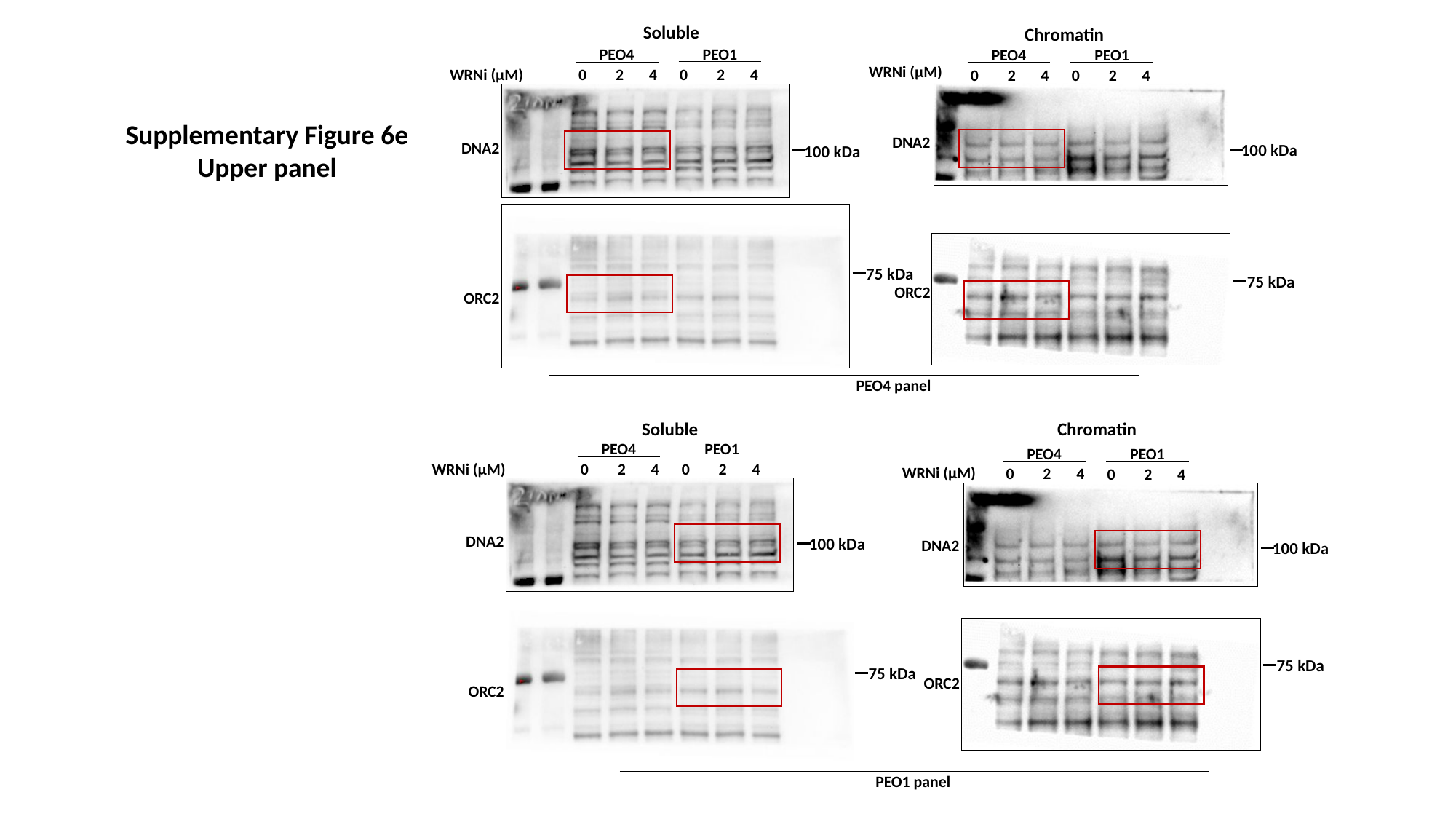

Soluble
Chromatin
PEO1
PEO4
PEO1
PEO4
WRNi (µM)
WRNi (µM)
0 2 4
0 2 4
0 2 4
0 2 4
Supplementary Figure 6e
Upper panel
DNA2
DNA2
100 kDa
100 kDa
75 kDa
75 kDa
ORC2
ORC2
PEO4 panel
Chromatin
Soluble
PEO1
PEO4
PEO1
PEO4
0 2 4
WRNi (µM)
0 2 4
WRNi (µM)
0 2 4
0 2 4
DNA2
100 kDa
DNA2
100 kDa
75 kDa
75 kDa
ORC2
ORC2
PEO1 panel

## Slide 16
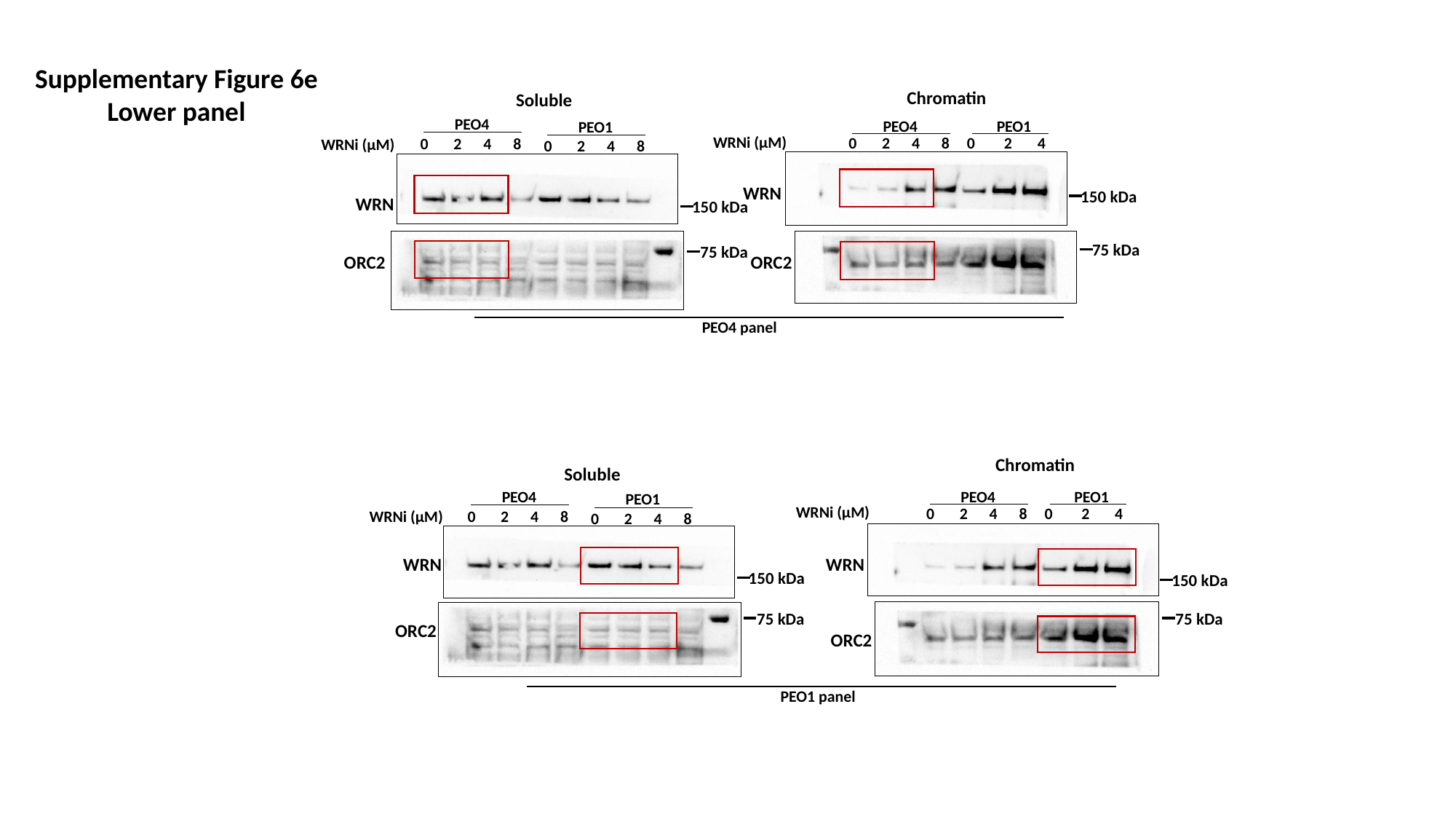

Supplementary Figure 6e
Lower panel
Chromatin
Soluble
PEO4
PEO4
PEO1
PEO1
WRNi (µM)
0 2 4
0 2 4 8
0 2 4 8
WRNi (µM)
0 2 4 8
WRN
150 kDa
WRN
150 kDa
75 kDa
75 kDa
ORC2
ORC2
PEO4 panel
Chromatin
Soluble
PEO4
PEO1
PEO4
PEO1
WRNi (µM)
0 2 4
0 2 4 8
0 2 4 8
WRNi (µM)
0 2 4 8
WRN
WRN
150 kDa
150 kDa
75 kDa
75 kDa
ORC2
ORC2
PEO1 panel

## Slide 17
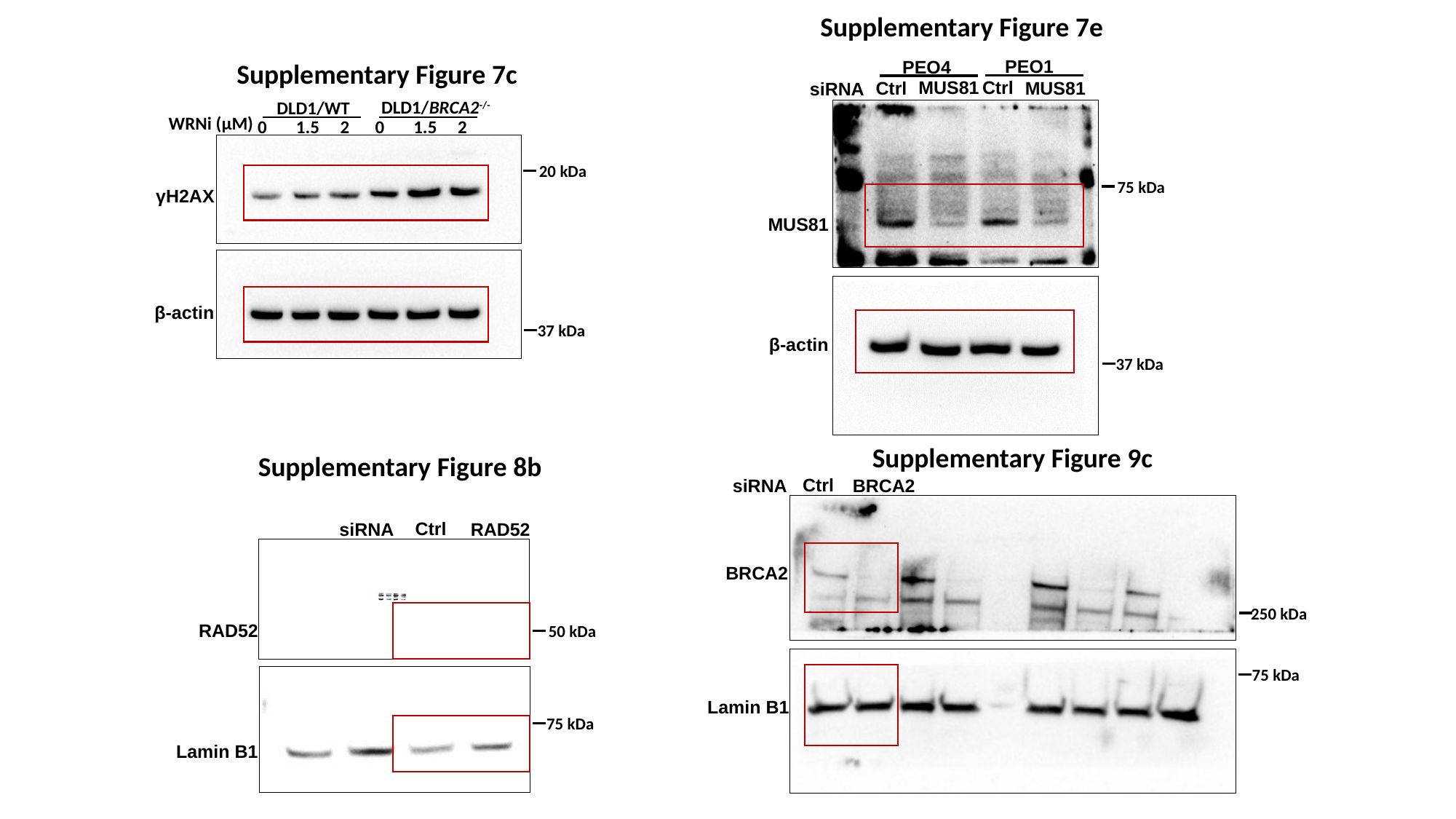

Supplementary Figure 7e
PEO1
PEO4
Supplementary Figure 7c
Ctrl
MUS81
Ctrl
MUS81
siRNA
DLD1/BRCA2-/-
DLD1/WT
WRNi (μM)
0 1.5 2
0 1.5 2
γH2AX
β-actin
20 kDa
75 kDa
MUS81
37 kDa
β-actin
37 kDa
Supplementary Figure 9c
Supplementary Figure 8b
Ctrl
siRNA
BRCA2
BRCA2
Lamin B1
Ctrl
siRNA
RAD52
RAD52
Lamin B1
250 kDa
50 kDa
75 kDa
75 kDa

## Slide 18
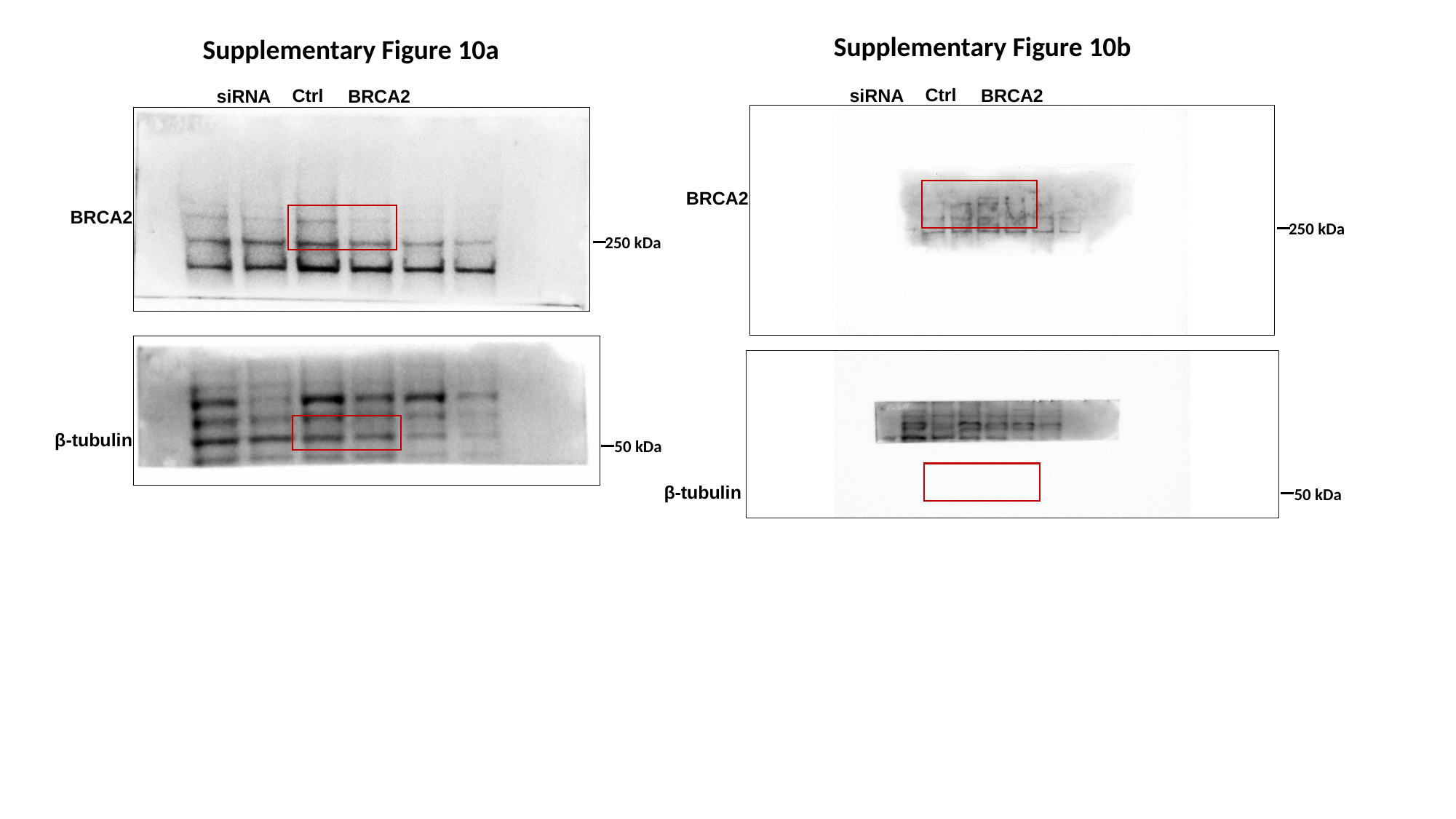

Supplementary Figure 10b
Supplementary Figure 10a
Ctrl
siRNA
Ctrl
BRCA2
siRNA
BRCA2
BRCA2
β-tubulin
BRCA2
β-tubulin
250 kDa
250 kDa
50 kDa
50 kDa

## Slide 19
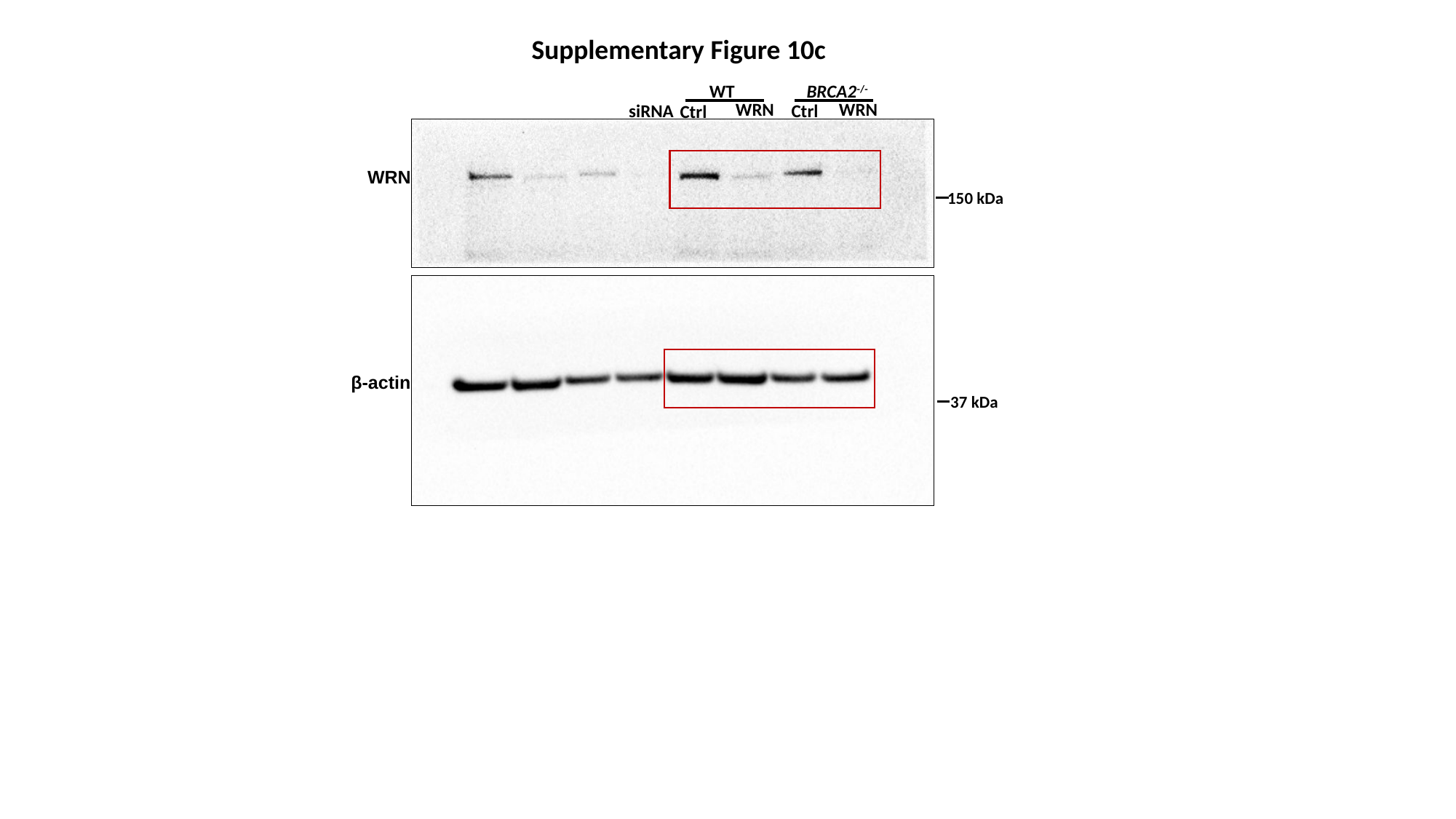

Supplementary Figure 10c
WT
BRCA2-/-
WRN
WRN
Ctrl
siRNA
Ctrl
WRN
β-actin
150 kDa
37 kDa

## Slide 20
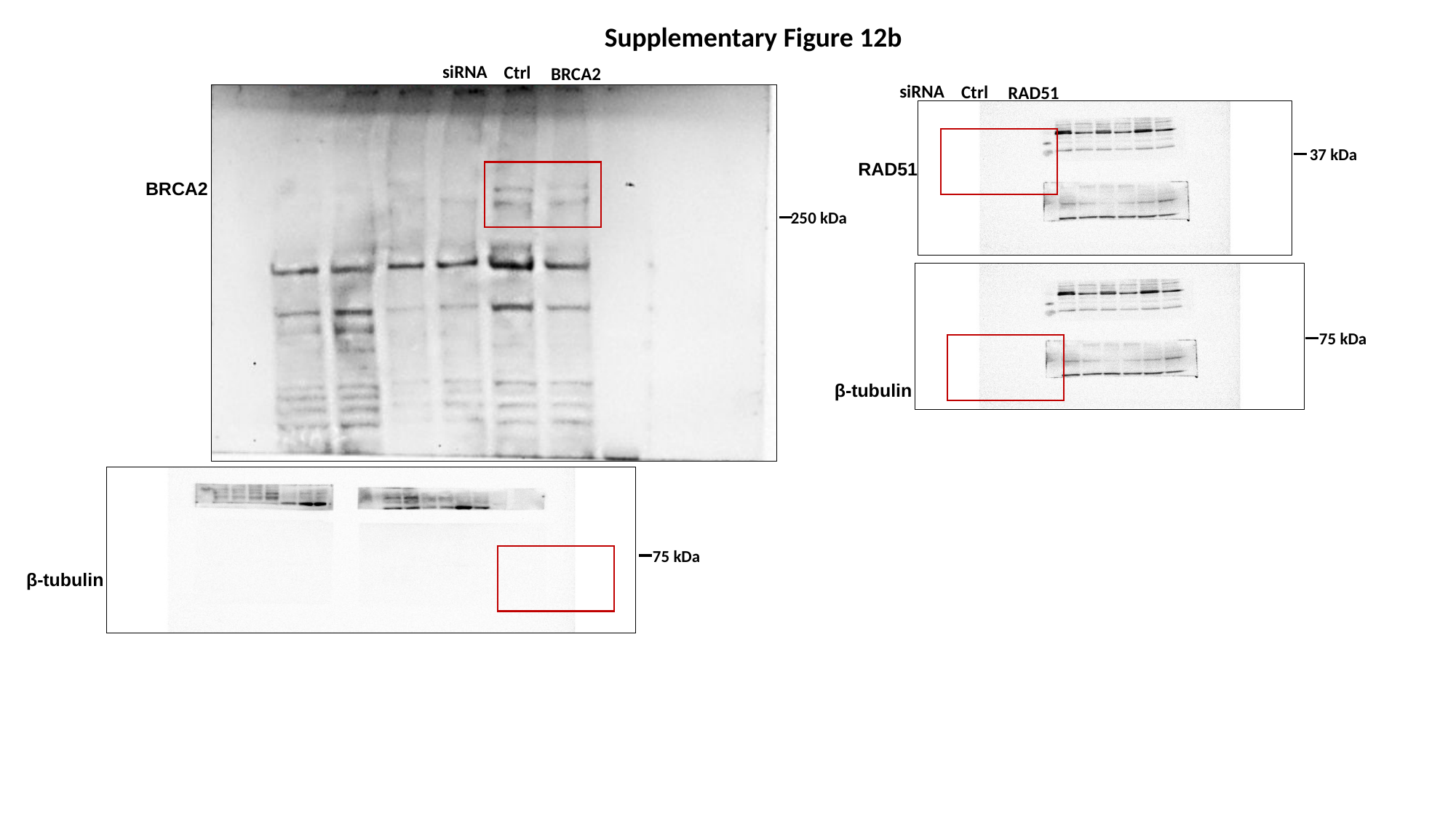

Supplementary Figure 12b
siRNA
Ctrl
BRCA2
siRNA
Ctrl
RAD51
BRCA2
β-tubulin
RAD51
β-tubulin
37 kDa
250 kDa
75 kDa
75 kDa

## Slide 21
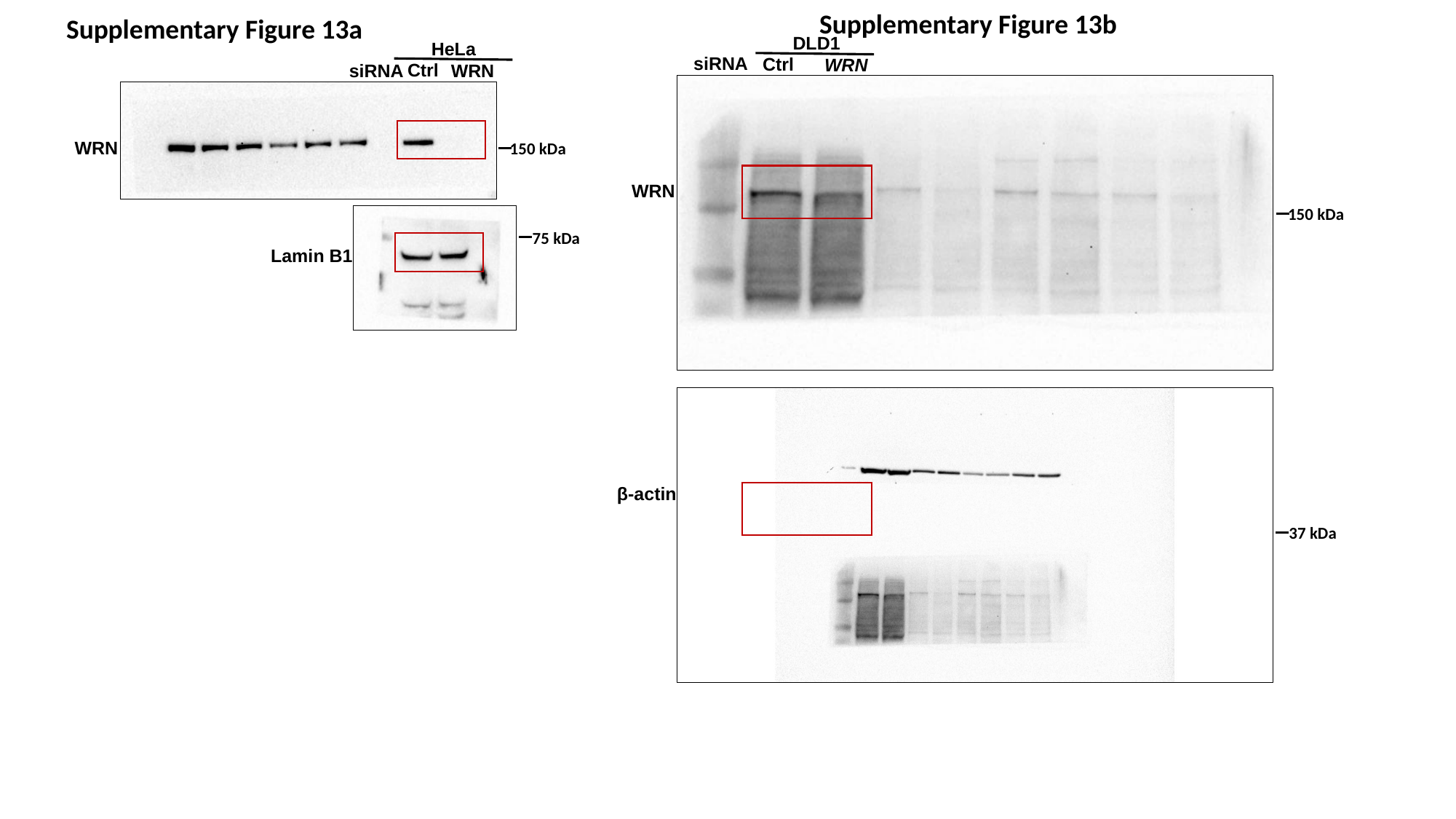

Supplementary Figure 13b
Supplementary Figure 13a
DLD1
HeLa
siRNA
 Ctrl
 WRN
Ctrl
siRNA
WRN
 WRN
β-actin
WRN
Lamin B1
150 kDa
150 kDa
75 kDa
37 kDa

## Slide 22
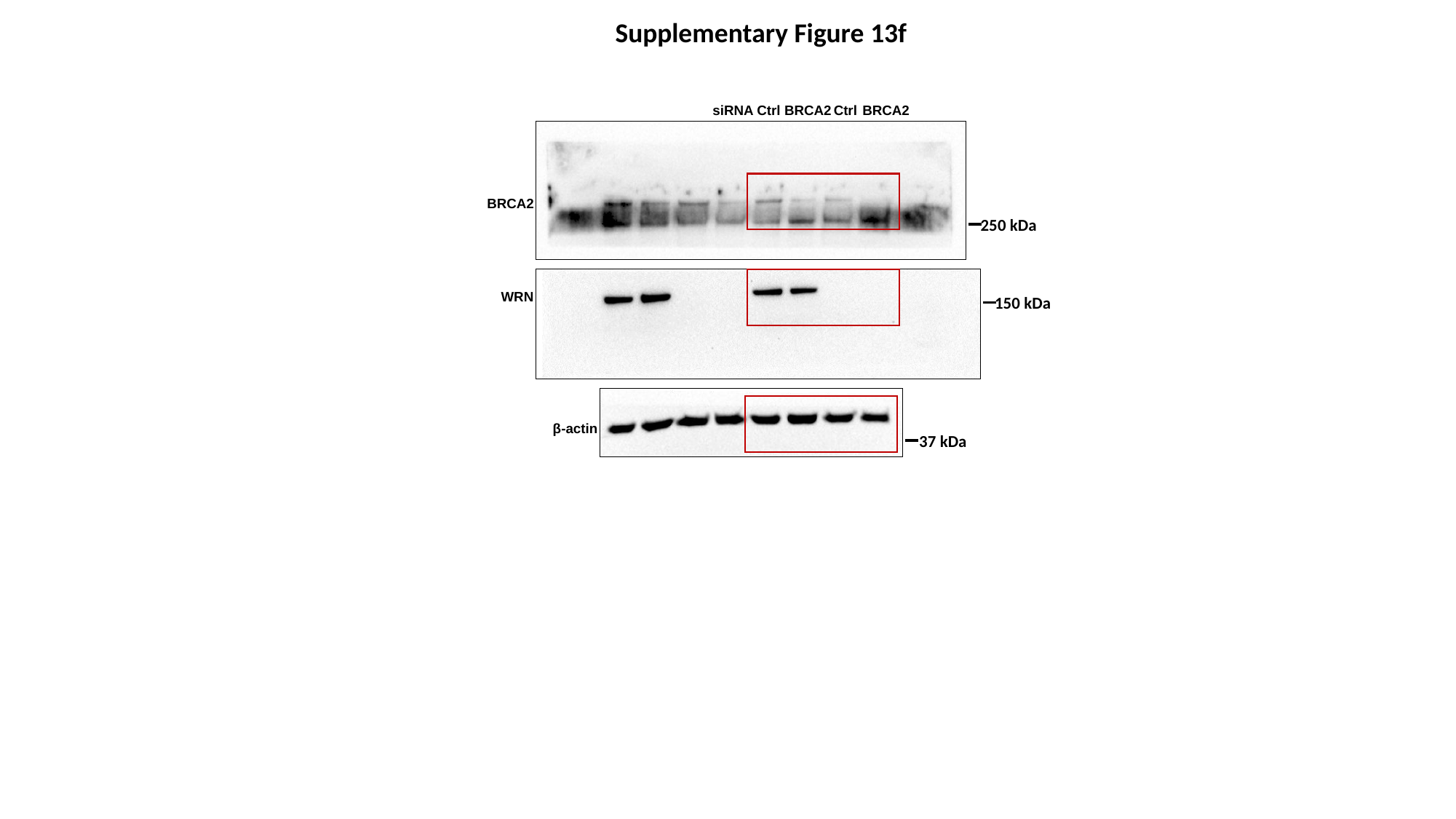

Supplementary Figure 13f
BRCA2
BRCA2
Ctrl
Ctrl
siRNA
BRCA2
WRN
β-actin
250 kDa
150 kDa
37 kDa
